# Tailoring thermoresponsiveness of biocompatible polyethers: copolymers of linear glycerol and ethyl glycidyl ether†

**DOI:** 10.1039/d3py00064h

**Published:** 2023-04-14

**Authors:** Verena Müller, Rebecca Matthes, Manfred Wagner, Matthias Bros, Philip Dreier, Holger Frey

**Affiliations:** a Department of Chemistry, Johannes Gutenberg University Duesbergweg 10-14 D-55128 Mainz Germany hfrey@uni-mainz.de; b Max Planck Institute for Polymer Chemistry Ackermannweg 10 D-55128 Mainz Germany; c University Medical Centre, Johannes Gutenberg University Langenbeckstraße 1 D-55101 Mainz Germany

## Abstract

Linear polyglycerol is known as a highly hydrophilic and biocompatible polymer that is currently considered for numerous medical applications. Derived from this well-known structure, the synthesis of highly biocompatible, thermoresponsive polyether copolymers *via* statistical anionic ring-opening copolymerization of ethyl glycidyl ether (EGE) and ethoxy ethyl glycidyl ether (EEGE) is described. Subsequent deprotection of the acetal groups of EEGE yields copolymers of linear glycerol (*lin*G) and EGE, P(*lin*G-*co*-EGE). These copolymers showed monomodal and narrow molecular weight distributions with dispersities *Đ* ≤ 1.07. The microstructure was investigated *via in situ*^1^H NMR kinetics experiments, revealing reactivity ratios of *r*_EEGE_ = 1.787 ± 0.007 and *r*_EGE_ = 0.560 ± 0.002, showing a slightly favored incorporation of EEGE over EGE. Due to the deliberate incorporation of rather hydrophobic EGE units into the water soluble *lin*PG, tunable thermoresponsive behavior is achieved with cloud point temperatures *T*_cp_ between 9.0–71.4 °C. Besides the commonly utilized method turbidimetry, temperature-dependent ^1^H NMR measurements were used for more accurate and reproducible results. The change of the hydrodynamic radii *r*_H_ of the copolymers and their aggregates upon reaching *T*_cp_ was investigated *via* DOSY NMR spectroscopy. To explore possible biomedical applications, as an example, the cell viability and immunology of an exemplary P(*lin*G-*co*-EGE) copolymer sample was investigated. Since both, cell viability and immunology are comparable to the gold standard PEG, the herein presented copolymers show high potential as biocompatible and thermoresponsive alternatives to PEG for biomedical applications.

## Abstract 2

Statistical copolymers of linear glycerol (*lin*G) and ethyl glycidyl ether (EGE) show tunable thermoresponsive behavior. The cloud point of the highly biocompatible copolymers is tailored by polymer composition and concentration.

## Graphical abstract

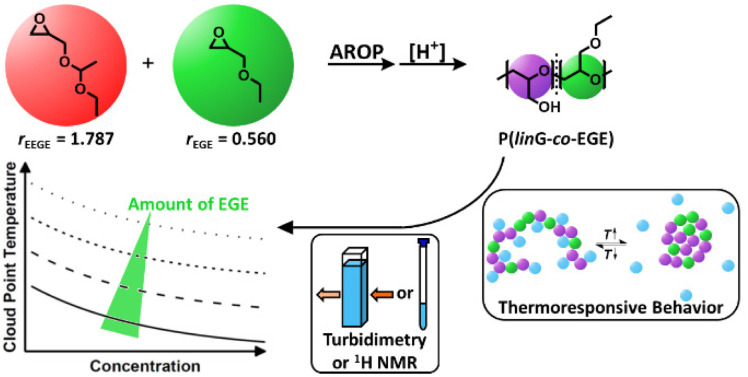

## Introduction

Polymers showing responsive behavior to external stimuli, especially thermoresponsive behavior, are promising materials for many applications, *e.g.* drug delivery^1,2^ or tissue engineering.^3,4^ Thermoresponsive polymers with a lower critical solution temperature (LCST) are water soluble due to strong hydration of the polymer molecules below a critical temperature, the cloud point temperature *T*_cp_. Upon temperature increase above the *T*_cp_, the polymer chains aggregate due to inter- and intramolecular interaction.^5,6^ Thermodynamically, the cloud point phase separation is driven by unfavorable entropy of mixing.^2,4,5^

The most intensely studied thermoresponsive polymer is poly(*N*-isopropylacrylamide) (PNIPAAm), which exhibits LCST behavior with *T*_cp_ = 32 °C.^7^ However, there is a controversial discussion regarding the toxicity of PNIPAAm. Monomer impurities in the polymer can cause cytotoxicity and therefore PNIPAAm has been viewed to be problematic for biomedical applications.^8^

Biocompatible alternatives to PNIPAAm may be found in the polymer class of thermoresponsive polyethers.^9^ Generally, aliphatic polyethers can be synthesized *via* anionic ring-opening polymerization (AROP),^10^ monomer-activated ring-opening polymerization (MAROP)^11^ or AROP with phosphazene bases^12–14^ amongst other (organo-)catalytic pathways. The AROP of glycidyl ethers is limited with respect to molecular weights due to proton abstraction in α-position of the epoxide moiety under the harsh, basic polymerization conditions.^15^ This side reaction, also known for the AROP of propylene oxide,^10^ can be prevented to some extent, if a weakly binding counterion like Cs^+^ is utilized in a polar and aprotic solvent at room temperature.^10,16^ Nevertheless, to obtain higher molecular weights, both AROP with phosphazene bases and MAROP were introduced. The drawback of both polymerization techniques is the demanding work-up procedure and the toxicity of impurities, *i.e.* phosphazene base or inorganic salt, which can cause cytotoxicity of the obtained polymers.^10–13,17^ If biomedical applications of polyethers are targeted, AROP is best suited and highly established for medical grade PEG, because no toxic catalyst is required, and highly defined materials are obtained.^18^

Aoki *et al.*^19^ investigated the thermoresponsive behavior of poly(glycidyl ether) homopolymers, namely poly(glycidyl methyl ether) (PGME) and poly(ethyl glycidyl ether) (PEGE) *via* turbidimetry and reported a *T*_cp_ of 57.7 °C and 14.6 °C, respectively. Schmalz *et al.*^20^ introduced statistical copolymers of glycidyl methyl ether (GME) and ethyl glycidyl ether (EGE) (P(GME-*co*-EGE)) with *T*_cp_ between 10 and 58 °C, depending on the copolymer composition. An increase of the amount of the more hydrophilic comonomer GME leads to an increasing *T*_cp_. The *T*_cp_ is not merely dependent on the copolymer composition, but equally on the polymer solution concentration and molecular weight.^21,22^ In a detailed study, Weinhart *et al.*^21^ synthesized random P(GME-*co*-EGE) copolymers and investigated the effect of increasing concentration and molecular weight on the *T*_cp_, both leading to decreased *T*_cp_.^5,21^ The presence of hydroxy functionalities instead of methoxy or ethoxy groups in each repeating unit results in the homopolymer of glycerol, linear polyglycerol (*lin*PG), which shows very high aqueous solubility and biocompatibility, surpassing even the current gold standard poly(ethylene glycol) (PEG).^23–25^ Since direct AROP of the corresponding monomer glycidol leads to hyperbranched polyglycerol, *lin*PG is commonly obtained *via* deprotection of various poly(glycidyl ethers), based on ethoxy ethyl glycidyl ether (EEGE), allyl glycidyl ether (AGE), *tert*-butyl glycidyl ether (*t*BGE) or benzyl glycidyl ether (BnGE).^24,26,27^ Due to the presence of the side chains and their atactic nature resulting from the polymerization of racemic monomer mixtures, *lin*PG is obtained as an amorphous material.^24^ In contrast, PEG, which is a biocompatible polyether used in medicine and pharmaceutics, possesses no side chains and represents a semi-crystalline polymer.^10,28^ As recently shown by Kakuchi *et al.*,^27^ the copolymerization of protected glycidol with a more hydrophobic comonomer relying on phosphazene-base promoted AROP can be exploited to tailor the LCST behavior of the resulting copolymers. The authors reported the synthesis of the copolymer P(*lin*G-*co*-EGE) and corresponding (multi)block copolymers. These P(*lin*G-*co*-EGE) copolymers showed thermoresponsive behavior in aqueous solution with *T*_cp_ values ranging between 30.5 and 70.4 °C, depending on the copolymer composition.

The thermoresponsive behavior of copolymers depends on the copolymer microstructure, which is governed by the polymerization technique. The reactivity ratios of the copolymerization of GME and EGE are very similar, when the polymerization is conducted *via* MAROP (*r*_GME_ = 0.98 and *r*_EGE_ = 0.95 (Kelen-Tüdõs method)^21^), albeit very different under AROP conditions (*r*_GME_ = 1.31 and *r*_EGE_ = 0.55 (Fineman-Ross method)^20^). The ideally random EGE/GME copolymers prepared *via* MAROP by Weinhart *et al.*^21^ exhibit a sharp decrease of transmittance with increasing temperature. In contrast, the decrease of transmission was broadened for copolymers synthesized *via* AROP, which exhibit a soft gradient in the microstructure. As the molecular weights of the copolymers are comparable (*M*_n_ = 2200 g mol^−1^ (MAROP) and *M*_n_ = 1800 g mol^−1^ (AROP)) and the ratio of EGE : GME is the same (3 : 1), this difference was assigned to the different copolymer microstructure.^21^ Kakuchi *et al.*^27^ investigated the difference in the thermoresponsive behavior of statistical and (multi)block copolymers of EGE and *lin*G. The statistical copolymer P(*lin*G-*co*-EGE) with an EGE-amount of 60% exhibited thermoresponsive behavior with *T*_cp_ = 50.5 °C. Both the triblock copolymer P(EGE-*b-lin*G-*b*-EGE) and the pentablock copolymer P(EGE-*b-lin*G-*b*-EGE-*b-lin*G-*b*-EGE) with an EGE amount of 70% and 60%, respectively, showed thermoresponsive behavior with similar cloud points. In contrast, the block copolymer P(*lin*G-*b*-EGE) with an EGE amount of 60% shows a two-step change in transmission. First the transmittance decreases with increasing temperature, before it increases again.

The commonly utilized method for characterization of thermoresponsive behavior is turbidimetry due to its simple implementation in a temperature-controlled UV/Vis spectrometer. The drawback of this method is that measurements can be influenced by multiple factors, *e.g.* heating rate, wavelength, stirring rate and the cuvette.^5^ Further, external factors like humidity or air bubbles may also influence the measurements. Other characterization methods for thermoresponsive behavior are dynamic light scattering (DLS),^5,29^ differential scanning calorimetry (DSC)^5,30^ or ^1^H NMR spectroscopy.^5,31^ The latter detects the thermoresponsive change in structure on a molecular level and is therefore highly precise.^5^

Here we describe the synthesis of thermoresponsive statistical P(*lin*G-*co*-EGE) copolymers *via* AROP of EEGE with EGE and subsequent removal of the acetal protective groups. Since the polymers are intended for biomedical purposes, the use of phosphazene bases is avoided, and “classical” AROP was employed. Molecular weights in the range of 3000 to 4500 g mol^−1^ were targeted, since this molecular weight range is often utilized in biomedical or pharmaceutical applications of PEG.^25^ Reactivity ratios are determined *via* precise *in situ*^1^H NMR kinetics measurements to elucidate the respective copolymer microstructure. An in-depth comparison of the critical solution behavior studied both *via* turbidimetry and ^1^H NMR spectroscopy is presented. Since no toxic catalysts or additives are required for the AROP, the synthesized copolymers are promising for biomedical applications. To evidence suitability for this field, the biocompatibility is demonstrated by cell viability and immunology assays with several murine cell types.

## Experimental

### Materials

All reagents were purchased from TCI, Sigma Aldrich, Acros Organics or VWR and used as received, unless otherwise stated. Ethyl glycidyl ether (EGE) was distilled before use. THF was flushed through basic Al_2_O_3_ to remove the stabilizer butylated hydroxytoluene (BHT). The synthesis of ethoxy ethyl glycidyl ether (EEGE) was performed according to a literature synthesis by Fitton *et al.*^32^ EGE and EEGE were dried over CaH_2_ before all polymerizations. Dowex® was activated with conc. aqueous HCl. Deuterated solvents were purchased from Deutero GmbH.

### Instrumentation

^1^H NMR spectra at 400 MHz and ^13^C NMR spectra at 100 MHz were recorded on a Bruker Advance II 400 and are referenced internally to residual proton signals of the deuterated solvent. *In situ*^1^H NMR kinetics studies were performed at 300 MHz on a Bruker Advance III HD 300, referenced internally to residual proton signals of the deuterated solvent. The graphic representation of the *in situ*^1^H NMR kinetics was achieved with NIREVAL software from Steube, Johann, Frey *et al.*^33^ For *T*_cp_ measurements, ^1^H NMR spectra at 500 MHz were recorded on a Bruker Advance III BR 500/51 and are referenced internally to residual proton signals of the deuterated solvent. The samples were brought to a specific temperature, and this temperature was kept constant for 30 min before a spectrum was measured. After that, the temperature was increased by 1 °C. DOSY measurements were recorded at 500 MHz on a Bruker III BR 500/51. SEC measurements were performed in DMF (flow rate: 1 mL min^−1^) with the internal standard toluene using an Agilent 1100 series with a HEMA 300/100/40 Å column cascade and RI detector. Calibration was carried out using poly(ethylene glycol) (PEG) standards provided by PSS. Matrix-assisted laser desorption and ionization time-of-flight mass spectroscopy (MALDI-ToF MS) measurements were performed on a Bruker Autoflex Max MALDI-ToF/ToF using *trans*-2-[3-(4-*tert*-butylphenyl)-2-methyl-2-propenyliden]malononitrile (DCTB) or α-cyano-4-hydroxycinnamic acid (HCCA) as a matrix and trifluoroacetic acid potassium salt (KTFA) or lithium chloride (LiCl) as a salt additive. UV/Vis transmittance measurements were performed with a Jasco V-630 UV/Vis spectrometer (*λ* = 600 nm, quartz cuvette, *d* = 10 mm). Measurements were performed in Milli-Q water at varying concentrations between 0.1 and 10.0 mg mL^−1^ at heating/cooling rates of 1.0 °C min^−1^. The normalized transmittance *vs.* temperature curve was fitted *via* sigmoidal fit. The cloud point temperature *T*_cp_ was defined as the temperature with 50% normalized transmittance.

### *In situ* ^1^H NMR kinetic studies and determination of reactivity ratios

The pre-dried initiator, the cesium salt of 2-benzyloxyethanol, was dissolved in DMSO-d_6_ and an aliquot was added to an NMR tube equipped with a Teflon stopcock. The monomers (24 mol% EEGE, 76 mol% EGE, total: 20 vol-%) were dried over CaH_2_ and added at −60 °C to the initiator solution. The solution was heated up to 25 °C right before the NMR measurement. All spectra were measured at 300 MHz with a time interval of 5 min between the spectra. The copolymerization ratios were determined *via* the non-terminal Jaacks method^34^ by the decreasing monomer proton signals at 2.70–2.71 ppm (EGE) and 2.74–2.75 ppm (EEGE). More details can be found in the ESI.† Due to the calculated copolymerization parameters, the microstructure of the copolymer was determined using NIREVAL software from Steube, Johann, Frey *et al.*^33^

### Investigation of immune cell viability and immunophenotype

Spleens were retrieved from C57BL/6 mice using a 40 μm cell strainer (Greiner Bio-One) to obtain a single cell suspension. Erythrocytes were lysed with a hypotonic lysis buffer. Spleen cells (4 × 10^6^ mL^−1^) were resuspended in IMDM culture medium supplemented with 5% fetal bovine serum, 100 U mL^−1^ penicillin, 100 μg mL^−1^ streptomycin, 50 μm β-mercaptoethanol and 2 mm l-glutamine and transferred into FACS tubes (500 μL). The copolymer or mPEG (*M* = 5000 g mol^−1^) was added (*c* = 1 and 10 μg mL^−1^) and samples were incubated overnight. After washing, the samples were incubated with fluorescence-labeled antibodies (CD-11b-SB600, CD11c-BV421, CD19-SB702, CD86-FITC, Ly6G-PE/eFl610, MHCII-APC, NK1.1-PE; all from Biolegend) and FVD-eFl780 (ThermoFisher) to discriminate viable/dead cells. Then, the samples were fixed in PBS containing 2 mm EDTA and 0.7% paraformaldehyde and analyzed in an Attune NxT flow cytometer (ThermoFisher). The spleen cell populations were identified *via* sequential gating.

### Synthesis of protected poly(ethoxy ethyl glycidyl ether-*co*-ethyl glycidyl ether) (P(EEGE-*co*-EGE))

#### General procedure

CsOH·H_2_O (0.9 eq.), dissolved in THF/Milli-Q water, and 2-benzyloxyethanol (1.0 eq.), dissolved in benzene, were mixed and the solvents were azeotropically removed at 60 °C. The initiator salt was dissolved in dry DMSO and cooled down to −78 °C. EEGE and EGE were added to the initiator salt solution under high vacuum. The reaction mixture was heated to 25 °C and stirred for at least 24 h. Subsequently, the reaction mixture was dissolved in CH_2_Cl_2_ and extracted against deionized water (3×) and brine (1×). The organic phase was dried over MgSO_4_ and the solvent was removed under reduced pressure. The copolymers were obtained as a colourless to light yellow viscous liquid. Yields: 44–99%. ^1^H NMR (DMSO-d_6_, 400 MHz): *δ* = 7.24–7.36 (m, 5H, *H*_arom_), 4.64 (d, OC*H*(CH_3_)O), 4.49 (s, 2H, PhC*H*_2_O), 3.38–3.60 (m, polyether backbone), 1.17 (d, OCH(C*H*_3_)O), 1.09 (t, OCH_2_C*H*_3_) ppm.

### Synthesis of deprotected poly(linear glycerol-*co*-ethyl glycidyl ether) (P(*lin*G-*co*-EGE))

#### General procedure

The corresponding copolymer P(EEGE-*co*-EGE) was dissolved in methanol. The ion exchange resin Dowex® was added to the reaction mixture. The reaction mixture was stirred at 25 °C for at least 20 h before it was filtrated. The filtrate was dried over MgSO_4_, and the solvent was removed under reduced pressure. The products were obtained as colourless to light yellow viscous liquids. Yields: 72–95%. ^1^H NMR (DMSO-d_6_, 400 MHz): *δ* = 7.24–7.36 (m, 5H, *H*_arom_), 4.45–4.60 (m, PhC*H*_2_O and CH_2_O*H*), 3.29–3.60 (m, polyether backbone), 1.09 (t, OCH_2_C*H*_3_) ppm.

## Results and discussion

### Synthesis of protected poly(ethoxy ethyl glycidyl ether-*co*-ethyl glycidyl ether) (P(EEGE-*co*-EGE)) *via* AROP

**Scheme 1:**
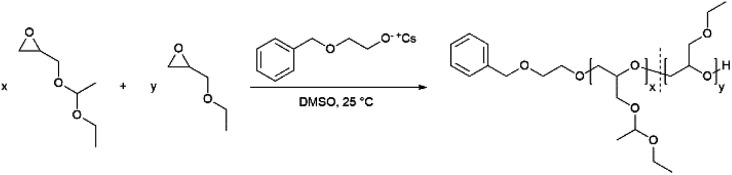
Synthesis of the copolymers P(EEGE-*co*-EGE) *via* AROP.

The monomer ethoxy ethyl glycidyl ether (EEGE) was synthesized from ethyl vinyl ether and glycidol according to an established protocol.^32^ The ^1^H NMR spectrum of the synthesized EEGE is shown in Fig. S1 (ESI).† For copolymerizations, the initiator 2-benzyloxyethanol was deprotonated with CsOH resulting in a degree of deprotonation of 90%. The copolymers with comonomer ratios between 10 : 90 and 80 : 20 mol% (EEGE : EGE) and the corresponding homopolymers were synthesized at room temperature (25 °C) in DMSO (Scheme 1). These polymerization conditions were crucial to suppress proton abstraction from the glycidyl ethers during polymerization.^10,16^ Compilations of all ^1^H NMR spectra and ^13^C NMR spectra are shown in Fig. S2 and S3 (ESI),† respectively. All signals can be assigned to the targeted copolymer structure. Importantly, there are no signals of allylic species (*δ* = 5.5–6.5 ppm), consequently no significant extent of proton abstraction was observed. The degrees of polymerization (*X*_n_) and copolymer compositions are listed in Table 1. The compositions were calculated from the corresponding ^1^H NMR spectra by comparison of the integrals of the methyl groups (CH_3_(5): *δ* = 1.17 ppm and CH_3_(4): *δ* = 1.19 ppm). The detailed calculation of the composition can be found in the ESI.† The determined compositions are in good agreement with the monomer ratios employed. Small deviations are caused by systematic errors during integration of the ^1^H NMR spectra.

**Table 1:** Composition of all P(EEGE-*co*-EGE) copolymers and the homopolymers PEGE and PEEGE

	Copolymer	*X*_n,th._ (EEGE : EGE)	*X*_n_ a (EEGE : EGE)	mol% EEGE_th._	mol% EEGE^a^
1	PEGE	0 : 30	0 : 37	0	0
2	P(EEGE_0.9_-*co*-EGE_0.91_)	3 : 27	3 : 31	10	9
3	P(EEGE_0.43_-*co*-EGE_0.57_)	10 : 15	11 : 14	40	43
4	P(EEGE_0.50_-*co*-EGE_0.50_)	13 : 13	11 : 11	50	50
5	P(EEGE_0.57_-*co*-EGE_0.43_)	18 : 12	18 : 13	60	57
6	P(EEGE_0.77_-*co*-EGE_0.23_)	24 : 6	24 : 7	80	77
7	PEEGE	22 : 0	25 : 0	100	100

a Determined by ^1^H NMR spectroscopy (400 MHz, DMSO-d_6_).

**Fig. 1:**
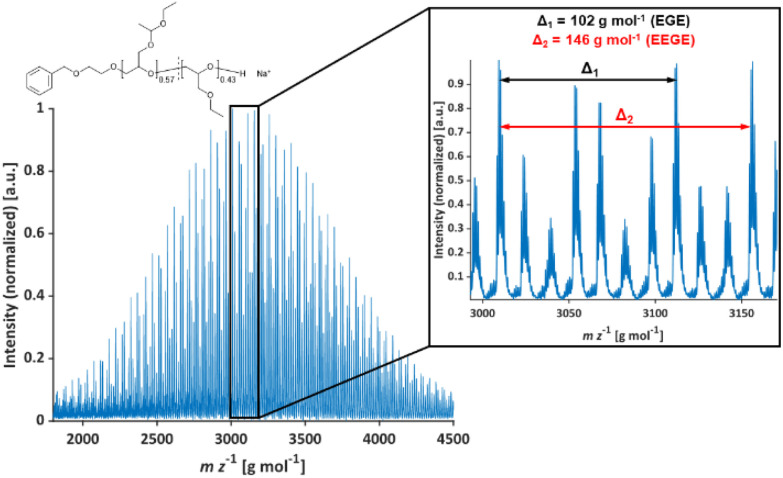
MALDI-ToF mass spectrum of P(EEGE_0.57_-*co*-EGE_0.43_); matrix: *trans*-2-[3-(4-*tert*-butylphenyl)-2-methyl-2-propenyliden]malononitrile (DCTB), salt additive: trifluoroacetic acid potassium salt (KTFA).

**Fig. 2:**
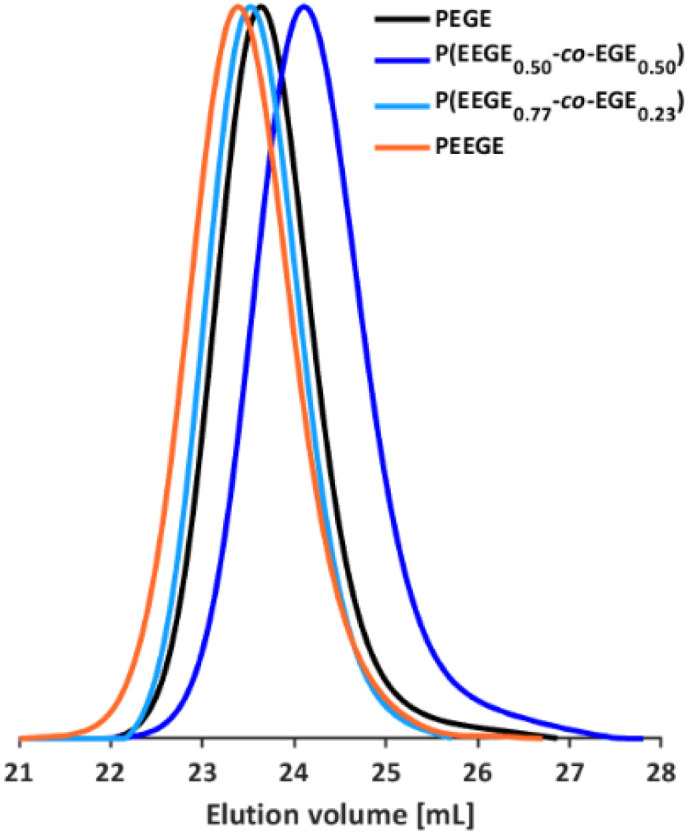
SEC curves (DMF, PEG calibration) of selected P(EEGE-*co*-EGE) copolymers and the homopolymers PEGE and PEEGE.

The molecular weights *M*_n_ of the copolymers were determined *via*^1^H NMR spectroscopy, SEC and MALDI-ToF MS, the dispersities *Đ* were determined by SEC. The targeted and determined molecular weights from ^1^H NMR spectroscopy are in good agreement, showing quantitative consumption of both monomers. The molecular weights determined by SEC are generally underestimated because of the deviating hydrodynamic radii of the copolymers. This is due to the presence of side chains and different polarity compared to the poly(ethylene glycol) (PEG) standards. All samples show narrow and monomodal molecular weight distributions in MALDI-ToF MS (Fig. 1) as well as in SEC (Fig. 2 and Fig. S5, ESI†), confirming the controlled copolymerization under the established polymerization conditions.

In Table 2, molecular weight characterization is presented. The lower *M*_n_ observed in MALDI-ToF MS measurements in comparison to ^1^H NMR measurements may be caused by partial deprotection of the acetal groups during the measurements (see Fig. S6, ESI†).

**Table 2:** Overview of characterization data of all P(EEGE-*co*-EGE) copolymers and the homopolymers PEGE and PEEGE

	Copolymer	*M*_n,th._ [g mol^−1^]	*M*_n_ a [g mol^−1^]	*M*_n_ b [g mol^−1^]	*M*_n_ c [g mol^−1^]	*Đ* b
1	PEGE	3400	3800	1900	2900	1.05
2	P(EEGE_0.9_-*co*-EGE_0.91_)	3300	3800	2000	3000	1.04
3	P(EEGE_0.43_-*co*-EGE_0.57_)	3100	2900	1700	2700	1.05
4	P(EEGE_0.50_-*co*-EGE_0.50_)	3400	2900	1500	2600	1.08
5	P(EEGE_0.57_-*co*-EGE_0.43_)	4000	4000	2000	3300	1.04
6	P(EEGE_0.77_-*co*-EGE_0.23_)	4300	4400	2000	3400	1.04
7	PEEGE	3500	3800	1800	3100	1.07

a Determined by ^1^H NMR spectroscopy.

b Determined by SEC.

c Determined by MALDI-ToF MS.

### Removal of protective groups to poly(linear glycerol-*co*-ethyl glycidyl ether) (P(*lin*G-*co*-EGE))

**Scheme 2:**
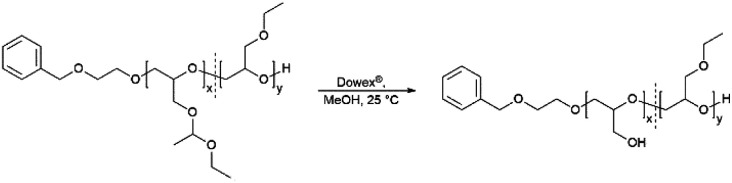
Synthesis of the copolymers P(*lin*G-*co*-EGE) *via* acidic deprotection with the ion exchange resin Dowex®.

**Fig. 3:**
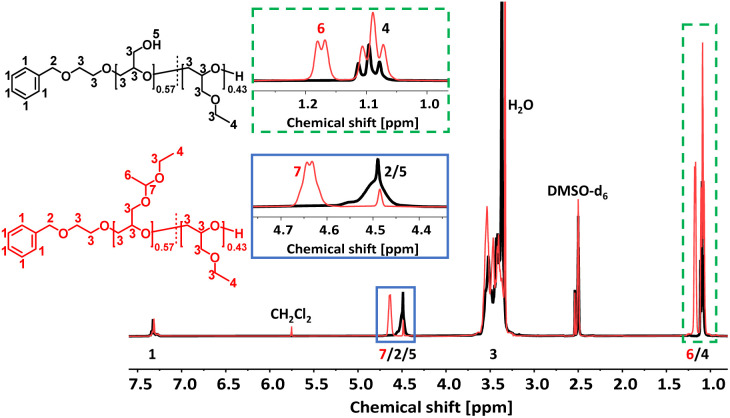
Stacked ^1^H NMR spectra (400 MHz, DMSO-d_6_) of P(*lin*G_0.57_-*co*-EGE_0.43_) (black) and P(EEGE_0.57_-*co*-EGE_0.43_) (red).

The acetal protecting group of the EEGE units of the homo- and copolymers were removed *via* acidic deprotection using the ion exchange resin Dowex® (Scheme 2). Successful deprotection was confirmed by ^1^H NMR spectroscopy, as shown exemplarily for the copolymer P(*lin*G_0.57_-*co*-EGE_0.43_) in Fig. 3. The ^1^H NMR spectrum confirms the absence of acetal protons (*δ* = 4.64 ppm) and protons of the methyl group next to the acetal group (*δ* = 1.17 ppm) after deprotection (black spectrum, Fig. 3), indicating the successful cleavage of the protecting groups. Additionally, the signal for the hydroxy group (*δ* = 4.50 ppm) after deprotection can be assigned to the deprotected *lin*G units. The ^1^H NMR and ^13^C NMR spectra for all copolymers are shown in Fig. S7 and S8 (ESI),† respectively.

**Fig. 4:**
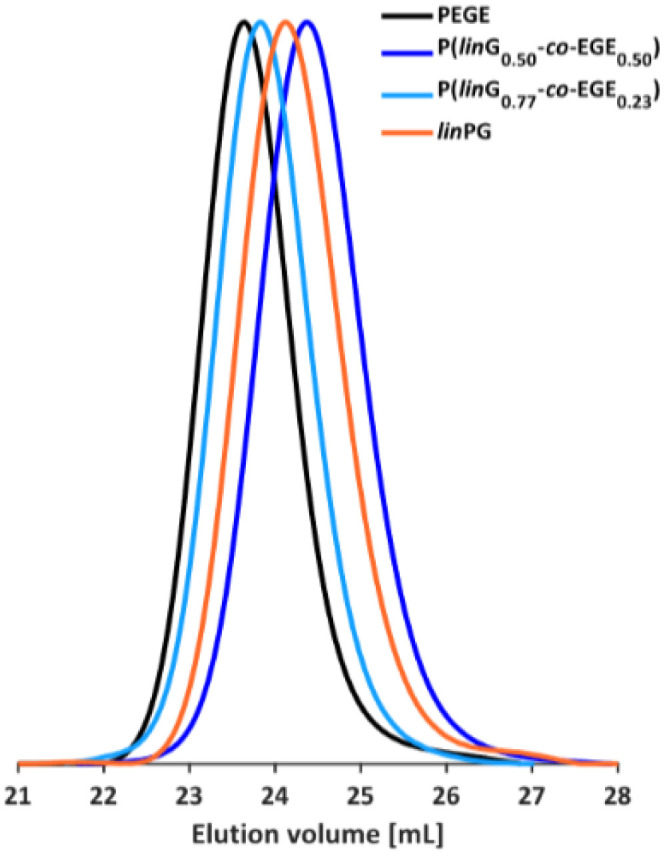
SEC curves (DMF, PEG calibration) of selected P(*lin*G-*co*-EGE) copolymers and the homopolymers PEGE and *lin*PG.

The molecular weights of the deprotected *lin*PG copolymers were analyzed in analogy to the aforementioned copolymer samples and are in good agreement with the calculated molecular weights. The molecular weight distributions determined by SEC (Fig. 4 and Fig. S9–S10, ESI†) are narrow and monomodal for all samples, with the exception of P(*lin*G_0.57_-*co*-EGE_0.43_). All MALDI-ToF mass spectra show monomodal molecular weight distributions (example in Fig. S11, ESI†). Table 3 compares the investigated molecular weights. Differences of the molecular weights of MALDI-ToF MS analysis compared to the calculated *M*_n_ are caused by the determination of the theoretical molecular weights, which is based on the error-prone integration of ^1^H NMR spectra.

**Table 3:** Overview of characterization data of all P(*lin*G-*co*-EGE) copolymers and the homopolymers PEGE and *lin*PG

	Copolymer	*M*_n,th._ a [g mol^−1^]	*M*_n_ b [g mol^−1^]	*M*_n_ c [g mol^−1^]	*M*_n_ d [g mol^−1^]	*Đ* b
1	PEGE	3400	3800	1900	2900	1.05
2	P(*lin*G_9_-*co*-EGE_91_)	3500	3300	1900	2900	1.06
3	P(*lin*G_43_-*co*-EGE_57_)	2200	2600	1600	1900	1.07
4	P(*lin*G_50_-*co*-EGE_50_)	2100	2200	1400	1600	1.07
5	P(*lin*G_57_-*co*-EGE_43_)	2800	2400	1900	2100	1.12
6	P(*lin*G_77_-*co*-EGE_23_)	2600	2300	1700	1800	1.06
7	*lin*PG	2000	2200	1500	1600	1.07

a Calculated from the composition of P(EEGE-*co*-EGE) copolymers assuming complete deprotection of the acetal protecting groups.

b Determined by ^1^H NMR spectroscopy.

c Determined by SEC.

d Determined by MALDI-ToF MS.

### Determination of the reactivity ratios of the monomers EEGE (*r*_EEGE_) and EGE (*r*_EGE_) polymerized by AROP

**Fig. 5:**
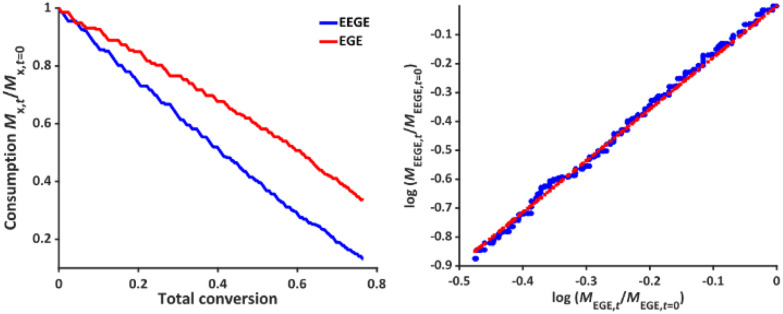
Left: Monomer consumption *M*_*x*,*t*_/*M*_*x*,*t* = 0_*versus* total conversion of P(EEGE_0.24_-*co*-EGE_0.76_), determined by *in situ*^1^H NMR kinetics study. Right: Logarithmic data fit based on the Jaacks equation to evaluate the reactivity ratios at 25 °C. Blue: Calculated data, red: linear fit. Reactivity ratios: *r*_EEGE_ = 1.787 ± 0.007, *r*_EGE_ = 0.560 ± 0.002 with a coefficient of determination *R*^2^ = 0.996.

To determine the reactivity ratios, the copolymerization of EEGE and EGE was performed in DMSO-d_6_ inside a NMR tube, employing the synthesis conditions described above. For these studies, the targeted composition was 24 mol% EEGE and 76 mol% EGE, respectively. For determination of the reactivity ratios, the consumption of both monomers was followed *via in situ*^1^H NMR spectroscopy. Due to similar chemical shifts of the monomer proton signals in the ^1^H NMR spectrum, the non-overlapping part of one proton of the epoxide methylene group of each monomer is considered exclusively (Fig. S12, ESI†). Fig. 5 (left) shows the consumption of each monomer *vs.* total conversion, demonstrating faster incorporation of EEGE compared to EGE. This is further evaluated by the determination of the reactivity ratios *r*_1_ and *r*_2_. The reactivity ratios are defined by the varying rate constants *k*_11_, *k*_12_, *k*_21_ and *k*_22_ (eqn (1) and (2)).1
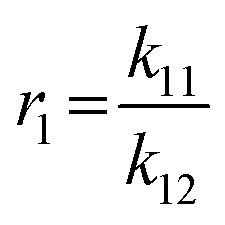
2
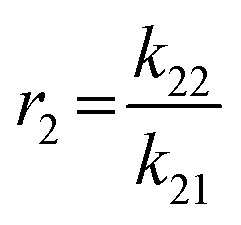


In non-terminal models, the reactivity ratio *r*_2_ is inversely proportional to *r*_1_ (*r*_2_ = *r*_1_^−1^), relying on the assumption that the reactivity of the chain end is independent of the nature of the active species (EEGE or EGE chain end). As a result, the relative incorporation rate of the monomers is merely dependent on the interaction of the monomer with the counter ion at the chain end.^35,36^

**Fig. 6:**
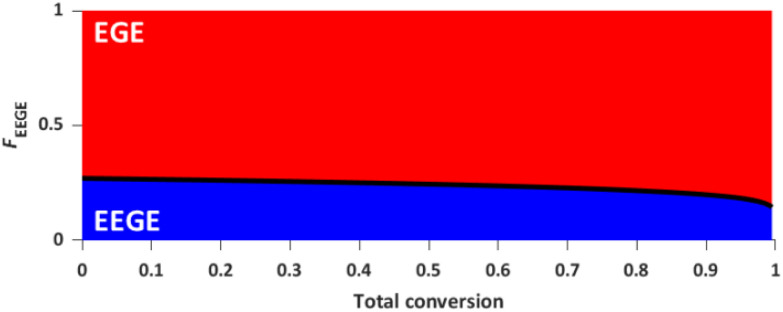
Monomer fraction *F versus* total conversion of P(EEGE_0.24_-*co*-EGE_0.76_).

The reactivity ratios of the copolymerization were calculated by the non-terminal Jaacks approach.^34^ From the Jaacks plot (Fig. 5, right), reactivity ratios of *r*_EEGE_ = 1.787 ± 0.007 and *r*_EGE_ = 0.560 ± 0.002 are obtained, mirroring a weak gradient microstructure of the copolymer. The microstructure of the copolymer is visualized by plotting the monomer fraction *F*_EEGE_*vs.* total conversion (Fig. 6). The slightly preferred incorporation of EEGE over EGE repeating units at the beginning of the copolymerization may be explained by the side chain of EEGE, which contain one additional oxygen atom compared to EGE. In analogy to the recently reported comparison of allyl glycidyl ether and ethoxy vinyl glycidyl ether, one additional oxygen atom increases the chelation capability of the glycidyl ether for the counter cation, leading to a slightly higher reactivity.^35,36^

### Lower critical solution temperatures (LCST)

#### Investigation of the critical solution behavior of P(EEGE-*co*-EGE) *via* turbidimetry

**Fig. 7:**
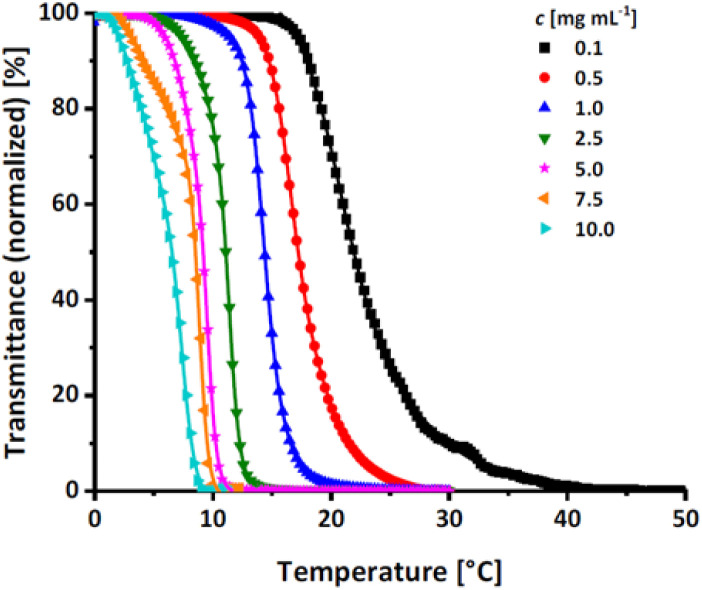
Transmittance *vs.* temperature plot of the copolymer P(EEGE_0.43_-*co*-EGE_0.57_) at different concentrations. Straight lines show sigmoidal fits to determine *T*_cp_ at 50% transmittance.

The effect of the hydrophobic, protected comonomer EEGE on the *T*_cp_ of PEGE was investigated for the copolymer P(EEGE_0.43_-*co*-EGE_0.57_) *via* UV/Vis spectroscopy at a wavelength of *λ* = 600 nm. The temperature dependent transmittance of the polymer solutions in Milli-Q water with concentrations between 0.1 and 10.0 mg mL^−1^ was examined. *T*_cp_ is defined as the temperature at 50% normalized transmittance, analyzed *via* a sigmoidal fit (sigmoidal function in ESI†). The transmittance *vs.* temperature plots are shown in Fig. 7, and Table 4 compares the determined *T*_cp_ with the homopolymer PEGE (see Fig. S13, ESI†). Due to the more hydrophobic comonomer EEGE, the *T*_cp_ of the copolymer P(EEGE_0.43_-*co*-EGE_0.57_) is decreased, compared to the *T*_cp_ of the homopolymer PEGE. The polymer-water interactions are less favored, therefore copolymer aggregation and precipitation occur at lower temperatures.

**Table 4:** Comparison of *T*_cp_ of the homopolymer PEGE and the acetal-protected copolymer P(EEGE_0.43_-*co*-EGE_0.57_)

Polymer	*c* [mg mL^−1^]						
	0.1	0.5	1.0	2.5	5.0	7.5	10.0
	*T*_cp_ [°C]						
P(EEGE_0.43_-*co*-EGE_0.57_)	21.9	17.2	14.4	11.1	9.2	8.5	6.5
PEGE	27.7	20.4	19.1	15.7	12.0	10.4	9.0

#### Investigation of the critical solution behavior of deprotected P(*lin*G-*co*-EGE) *via* turbidimetry

**Fig. 8:**
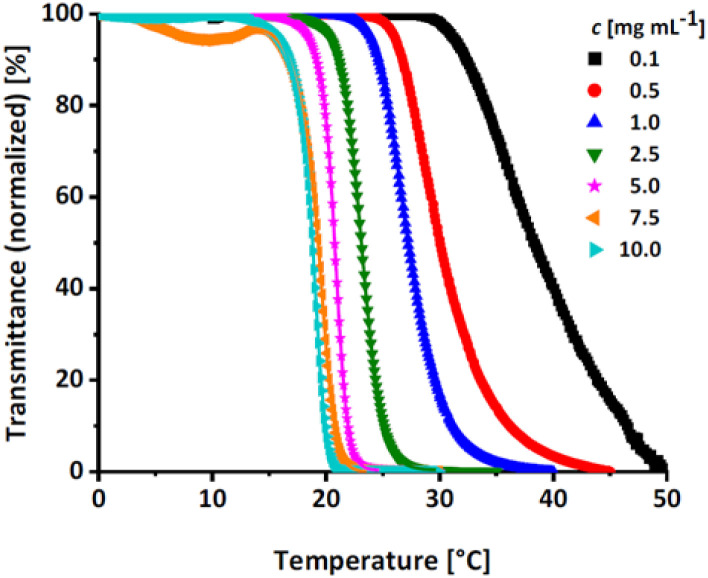
Transmittance *vs.* temperature plot of the copolymer P(*lin*G_0.09_-*co*-EGE_0.91_) at different concentrations. Straight lines show sigmoidal fits to determine *T*_cp_ at 50% transmittance.

Linear polyglycerol is a highly hydrophilic polymer^24^ that shows no change in transmission in the measurable temperature region (see Fig. S14, ESI†). To evaluate the effect of the rather apolar EGE moieties along the chains, the respective *T*_cp_ of all synthesized P(*lin*G-*co*-EGE) copolymers were determined after removal of the acetal protective groups. Fig. 8 shows the transmittance *vs.* temperature plots upon heating at the example of P(*lin*G_0.09_-*co*-EGE_0.91_). The corresponding plots for copolymers with other compositions (Table 3) are shown in Fig. S15 and S16 (ESI).† Increasing polymer concentration leads to a decrease of *T*_cp_. The probability of polymer–polymer interactions increases with concentration, which leads to the favored formation of polymer aggregates at lower temperatures. The change in transmittance is sharp for higher concentrations and is slightly broadened with decreasing concentrations. This is due to a lower local polymer concentration and therefore a more gradual collapse of the polymer chains at lower concentrations.^5^

**Fig. 9:**
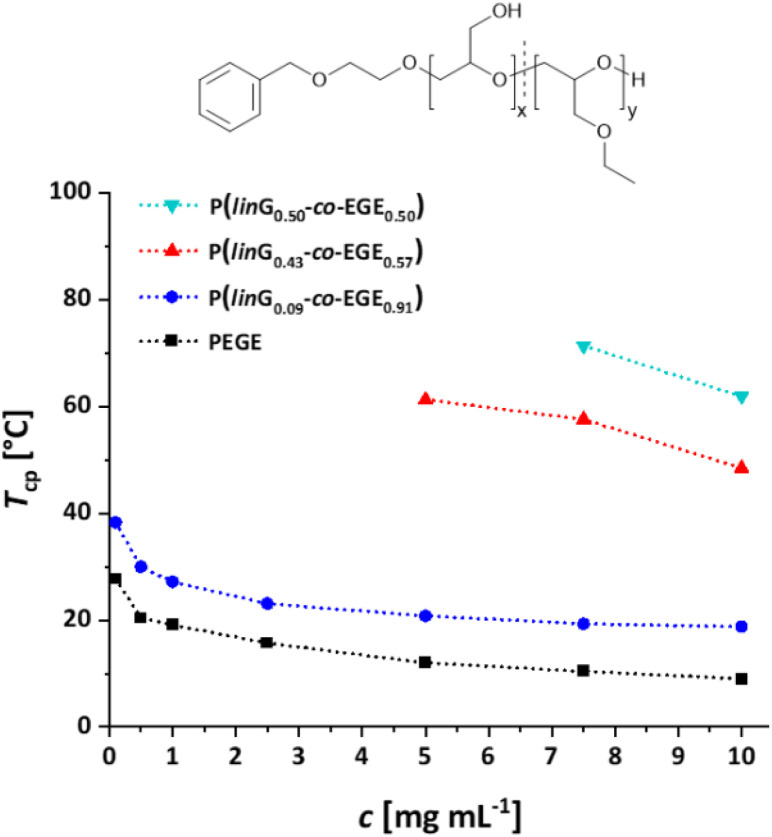
Summary of concentration dependent *T*_cp_ of P(*lin*G-*co*-EGE). Dotted lines are depicted to guide the eye.

Fig. S17 (ESI)† compares the heating and cooling curves for the copolymer P(*lin*G_0.09_-*co*-EGE_0.91_) as an example, at a concentration of *c* = 2.5 mg mL^−1^. Since the hysteresis is negligibly small, only the heating curves are taken into account hereafter. Table 5 and Fig. 9 summarize the *T*_cp_ of all P(*lin*G-*co*-EGE) copolymers and both homopolymers. There is a linear correlation of the content of *lin*G units with the increase of *T*_cp_. As expected, an increasing amount of *lin*G units enhances the polarity of the copolymers. Consequently, water-polymer interactions become favored due to hydrogen bonding between water molecules and the hydroxy functionalities. The copolymer with 43% *lin*G units shows no change in transmittance over the whole temperature range for *c* = 0.1 and an incomplete decrease of transmittance for *c* = 0.5–2.5 mg mL^−1^. If the content of *lin*G units exceeds 57%, no *T*_cp_ was observed for all concentrations.

**Table 5:** Overview of *T*_cp_ of all P(*lin*G-*co*-EGE) copolymers and homopolymers in aqueous solution

mol% *lin*G		*c* [mg mL^−1^]						
		0.1	0.5	1.0	2.5	5.0	7.5	10.0
		*T*_cp_ [°C]						
1	0	27.7	20.4	19.1	15.7	12.0	10.4	9.0
2	9	38.3	30.0	27.2	23.1	20.8	19.3	18.8
3	43	—	*	*	*	61.3	57.6	48.5
4	50	—	*	*	*	*	71.4	61.9
5	57	—	—	—	—	—	—	—
6	77	—	—	—	—	—	—	—
7	100	—	—	—	—	—	—	—

The described results regarding the thermoresponsive solution behavior of the presented copolymers with *lin*G amounts up to 50% are in line with the recent results of Kakuchi *et al.*^27^ These authors synthesized P(*lin*G-*co*-EGE) copolymers *via* a different route, capitalizing on phosphazene base-catalyzed AROP of EGE and benzyl glycidyl ether, followed by deprotection of the benzyl glycidyl ether *via* hydrogenation. However, above a *lin*G amount of 50%, Kakuchi *et al.* still observed *T*_cp_ values, while our copolymer samples exhibit full solubility up to 100 °C. This may be caused by the higher molecular weights of the copolymers reported by Kakuchi *et al.* (Kakuchi: 11–16 kg mol^−1^, compared to the herein presented copolymers with 2–4 kg mol^−1^) and therefore higher local concentration of copolymer in solution. This leads to an earlier aggregation of the copolymers because of a higher possibility for intra- and intermolecular polymer–polymer interactions and thus to a decrease of *T*_cp_.^21^ Further reasons for the differences in *T*_cp_ may lie in the microstructure of the copolymers. While the herein presented copolymers exhibit an almost ideally statistical comonomer distribution (Fig. 6), the microstructure of the copolymers of the Kakuchi group was not investigated, but likely deviates from the copolymers of this work due to the different monomer combination and copolymerization technique employed.

#### Investigation of the critical solution behavior of P(*lin*G-*co*-EGE) *via*^1^H NMR spectroscopy

**Fig. 10:**
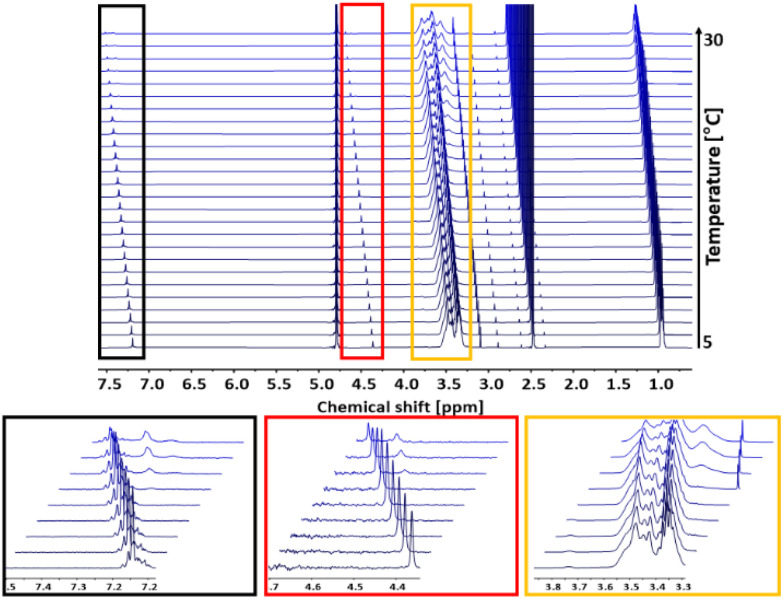
^1^H NMR analysis (500 MHz, D_2_O) of *T*_cp_ of the copolymer P(*lin*G_0.09_-*co*-EGE_0.91_) in a temperature range of 5–30 °C. Insets: Every third spectrum is shown for overview purposes.

Turbidimetry is a simple and efficient method to determine the critical solution behavior of polymers, but is also sensitive to external influences like humidity or dust impurities. Further, turbidimetry measurements may show a larger systematic error due to other parameters like for example wavelength, cuvette material or stirring rate. Compared to turbidimetry, ^1^H NMR spectroscopy directly follows the mobility of the polymer chains in solution and mirrors changes in the chemical and electronic environment of the polymer chains. An increase in temperature above *T*_cp_ and the resulting aggregation of the polymer chains is directly correlated with a decrease in their mobility.^5^ Hence, to support the turbidimetry measurements, temperature dependent ^1^H NMR measurements of the copolymer P(*lin*G_0.09_-*co*-EGE_0.91_) were carried out in D_2_O with a concentration of *c* = 10 mg mL^−1^. In between each measurement, the temperature was increased by 1 °C. The spectra of P(*lin*G_0.09_-*co*-EGE_0.91_) are shown in Fig. 10. The intensity of the polymer signals starts to decrease upon reaching *T*_cp,NMR_ = 19 °C as a result of chain aggregation. The mobility of these aggregates is strongly lowered compared to the free polymer chains. This decreases the transversal relaxation time *T*_2_, which leads to a broadening of the signals.^37^ Additionally, new signals occur at *T*_cp,NMR_ with chemical shifts to lower fields (see Fig. 10, insets). These new signals are ascribed to the structural change during the aggregation, because the electronic environment of the protons of the polymer chains changes.

In the following, *T*_cp,NMR_ defines the temperature at which polymer chains start to aggregate. To compare the results of the ^1^H NMR measurements with the turbidimetry experiment, *T*_cp,UV/VIS_ is herein defined as the temperature with 95% transmittance because this value marks the beginning of polymer chain aggregation. Compared to *T*_cp,NMR_ = 19 °C, *T*_cp,UV/Vis_ has a slightly lower value (*T*_cp,UV/Vis_ = 15.7 °C). This difference is possibly caused by applying different heating rates for each measurement. While the temperature was increased constantly with a heating rate of 1 °C min^−1^ in the turbidimetry measurement, the temperature in the ^1^H NMR analysis was kept constant for 30 min before each measurement. Hence, thermodynamic equilibrium between the free polymer chains and the polymer aggregates is established. Considering the precise and constant temperature setting as well as the less systematic error-prone set-up of the ^1^H NMR compared to UV/Vis analysis, the evaluation *via* NMR constitutes the preferable method leading to more reliable results for the determination of *T*_cp_.

#### Determination of the hydrodynamic radius *r*_H_ of P(*lin*G-*co*-EGE) *via* DOSY

**Fig. 11:**
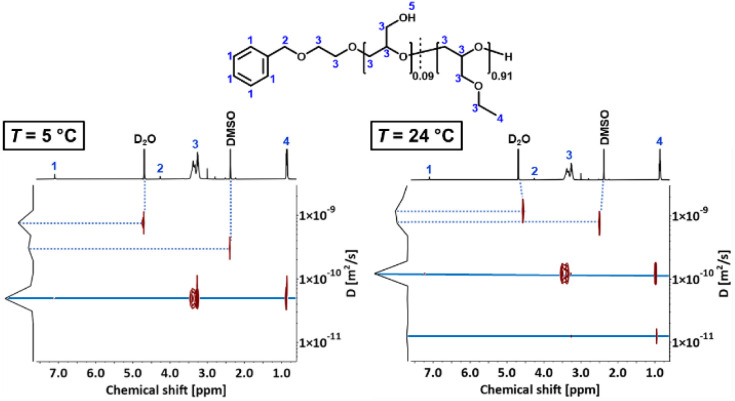
DOSY spectra of P(*lin*G_0.09_-*co*-EGE_0.91_) at *T* = 5 °C (*T* < *T*_cp_) and 24 °C (*T* > *T*_cp_).

To verify aggregation of the copolymer P(*lin*G-*co*-EGE) above *T*_cp_, the hydrodynamic radius of the copolymer P(*lin*G_0.09_-*co*-EGE_0.91_) and its aggregates was determined by DOSY NMR for both *T* < *T*_cp_ and *T* > *T*_cp_*via* the Stokes–Einstein equation (eqn (3)):^38^3
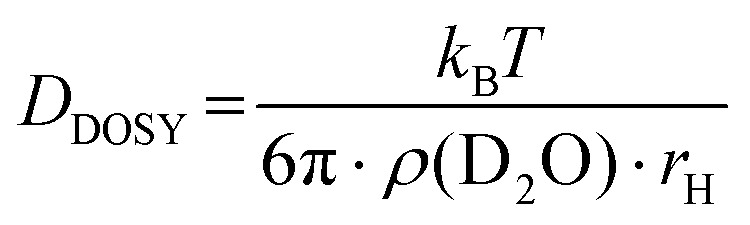
where *D*_DOSY_ is the diffusion coefficient determined *via* DOSY, *k*_b_ is the Boltzmann constant and *ρ*(D_2_O) is the viscosity of D_2_O at the measured temperature. The DOSY spectra for *T* < *T*_cp_ and *T* > *T*_cp_ are shown in Fig. 11, the corresponding diffusion coefficients and calculated hydrodynamic radii are listed in Table 6. If the temperature is below *T*_cp_, a single diffusion coefficient is detected, resulting in a corresponding hydrodynamic radius *r*_H_ of 2.1 nm. Above *T*_cp_, a second diffusion coefficient appears, which indicates that a fraction of the polymer chains already aggregates, while other parts remain dissolved in solution. The calculated hydrodynamic radii are *r*_H_ = 1.6 and 16.0 nm. The species with *r*_H_ = 1.6 nm relates to the dissolved copolymer chains in solution. The hydrodynamic radius decreases compared to the hydrodynamic radius at *T* < *T*_cp_. Above the cloud point temperatures, polymer–polymer interactions are preferred regarding an increase of entropy. Therefore, the copolymer collapses to reduce the interface to D_2_O. The second hydrodynamic radius (*r*_H_ = 16.0 nm) indicates polymer aggregates which are formed once *T*_cp_ is reached.

**Table 6:** Overview of diffusion coefficients and hydrodynamic radii of P(*lin*G_0.09_-*co*-EGE_0.91_) for *T* < *T*_cp_ and *T* > *T*_cp_. Viscosities ρ(D_2_O) were taken from literature^39^

*T* [°C]	*D* [10^−11^ m^2^ s^−1^]	*ρ*(D_2_O) [mPas]	*r*_H_ [nm]
5 (*T* < *T*_cp_)	4.882	1.988	2.1
24 (*T* > *T*_cp_)	12.337	1.100^a^	1.6
	1.236	1.100^a^	16.0

a Viscosity of D_2_O for *T* = 25 °C.^39^

### Immune cell viability and immunophenotype of P(*lin*G-*co*-EGE)

**Fig. 12:**
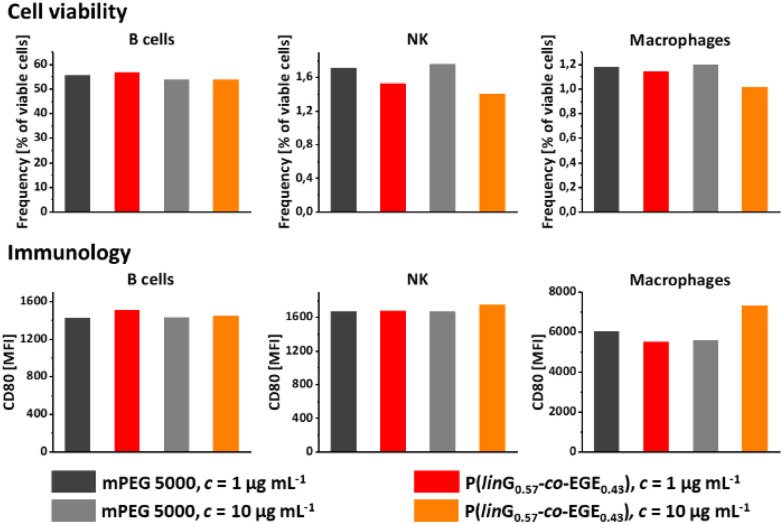
Cell viability (top) and immunology using the surface protein CD80 (MFI = mean fluorescence intensity) (bottom) of P(*lin*G_0.57_-*co*-EGE_0.43_) and mPEG (*M*_n_ = 5000 g mol^−1^) as a reference for B cells, natural killer cells (NK) and macrophages.

Both *lin*PG and copolymers of short chain alkyl glycidyl ethers (SCAGEs), *e.g.* copolymers of EGE and glycidyl methyl ether (GME) P(EGE-*co*-GME), are known for their excellent biocompatibility and high cell viability.^24,40^ To explore potential biomedical applications of the herein synthesized copolymer series P(*lin*G-*co*-EGE), the cell viability and effects of the synthesized copolymer sample P(*lin*G_0.57_-*co*-EGE_0.43_) as a typical representative of the copolyether series on immune cells were explored. For this purpose, the copolymer and monomethyl poly(ethylene glycol) (mPEG) (*M*_n_ = 5000 g mol^−1^) as a reference were incubated with murine cells (*c* = 1 and 10 μg mL^−1^) over night. The cell viability was investigated *via* fluorescence-activated cell sorting (FACS) for the following cell types: B cells, natural killer cells (NK), macrophages (Fig. 12, top), dendritic cells (DC), polymorphonuclear cells (PMN) and T cells (Fig. S18, top, ESI†). The copolymer P(*lin*G_0.57_-*co*-EGE_0.43_) shows comparable cell viability as the gold standard mPEG for all cell types for both concentrations, even at a higher concentration of *c* = 10 μg mL^−1^. Immunological behavior was investigated by expression of the surface proteins CD80 (Fig. 12, bottom and Fig. S18,† bottom), CD86 and MCHII (both Fig. S19, ESI†). The corresponding fluorescence labelled antibodies of these surface activation markers were measured by FACS. For all cell types, the immune cell activation of the copolymer is comparable to mPEG, independent of the investigated polymer concentration range. Hence, the results indicate the suitability of the presented copolymers for biomedical and pharmaceutical applications as a thermoresponsive alternative to mPEG.

## Conclusions

Statistical copolymers of ethyl glycidyl ether (EGE) and linear glycerol (*lin*G) with systematically varied comonomer composition have been prepared. For this purpose, EGE and ethoxy ethyl glycidyl ether (EEGE) were copolymerized *via* anionic ring-opening polymerization (AROP) at room temperature and subsequent acidic deprotection of the acetal group of EEGE. Molecular weights in the range of 2200 to 3800 g mol^−1^ were targeted and confirmed *via*^1^H NMR spectroscopy and MALDI ToF MS. The use of catalysts or monomer-activation was avoided to generate copolymers suitable for medical applications. SEC traces of both homopolymers (*lin*PG and PEGE) and copolymers (P(*lin*G_*x*_-*co*-EGE_1−*x*_), *x*: mol% *lin*G) showed very narrow, monomodal distributions (*Đ* = 1.04–1.07), confirming controlled homo- and copolymerization, respectively. The copolymerization kinetics of EGE and EEGE was investigated *via in situ*^1^H NMR kinetics measurements. Evaluation with the non-terminal Jaacks method resulted in reactivity ratios of *r*_EEGE_ = 1.787 ± 0.007 and *r*_EGE_ = 0.560 ± 0.002, confirming a slightly faster incorporation of EEGE over EGE in the polymer chains during AROP. These values show a slight gradient, evidencing an almost ideally random copolymer formation. Further, the thermoresponsive behavior of one exemplary P(EEGE-*co*-EGE) copolymer and all deprotected P(*lin*G-*co*-EGE) copolymers and homopolymers in aqueous solution was investigated *via* turbidimetry. Depending on the polarity (acetal or hydroxymethylene group) and the incorporated amount of side chains, the hydrophilicity of the copolymers can be tailored in a linear fashion. The *T*_cp_ of the copolymer P(*lin*G_0.09_-*co*-EGE_0.91_) was further investigated *via* temperature-dependent ^1^H NMR spectroscopy. Compared to the *T*_cp_ determined *via* turbidimetry, the *T*_cp_ of the ^1^H NMR measurements is slightly higher (Δ*T*_cp_ = 3 °C). This discrepancy might be caused by different heating rates and a less error-prone set-up of the ^1^H NMR measurement. In addition, the hydrodynamic radius *r*_H_ of the copolymer P(*lin*G_0.09_-*co*-EGE_0.91_) was investigated *via* DOSY NMR spectroscopy for both *T* < *T*_cp_ and *T* > *T*_cp_. For *T* < *T*_cp_ merely one species with *r*_H_ = 2.1 nm is observed. Above *T*_cp_, a second mode with a larger *r*_H_ of 16.0 nm appears, which can be assigned to aggregated copolymer chains. Cell viability and immunology of the synthesized copolymers were investigated for P(*lin*G_0.77_-*co*-EGE_0.23_) as a typical copolymer sample. Both cell viability and immunological properties are fully comparable to mPEG, the gold standard polyether broadly used in medicine and pharmaceutics.^28^ The herein presented copolymers are therefore suitable as a thermoresponsive alternative for mPEG in (bio)medical applications, permitting to tailor the LCST, *e.g.*, for nanomedicine and thermoresponsive therapeutics.

## Author contributions

The manuscript was written through contributions of all authors. All authors have given approval to the final version of the manuscript.

## Ethical statement

C57BL/6 mice were kept in the Central Animal Facility of the Johannes Gutenberg-University Mainz under pathogen-free conditions on a standard diet according to the guidelines of the regional animal care committee (Rhineland-Palatinate, Germany). The “Principles of Laboratory Animal Care” (NIH publication no. 85-23, revised 1985) were followed. Ethical review and approval were waived for this study due to exclusive use of isolated mouse cells derived from mice sacrificed for organ retrieval according to § 4(3) TierSchG.

## Conflicts of interest

There are no conflicts to declare.

## Supplementary Material

PY-014-D3PY00064H-s001
