# Supplementary material for: Tailoring thermoresponsiveness of biocompatible polyethers: copolymers of linear glycerol and ethyl glycidyl ether
Source: Polym Chem. 2023 Apr 14;14(21):2599–609. doi: 10.1039/d3py00064h (PMC10228176; doi:10.1039/d3py00064h)
Supplement: PY-014-D3PY00064H-s001 [file PY-014-D3PY00064H-s001.pdf]

Electronic Supplementary Material (ESI) for Polymer Chemistry.
This journal is © The Royal Society of Chemistry 2023

Supporting Information

Tailoring Thermoresponsiveness of Biocompatible Polyethers: Copolymers of Linear Glycerol and Ethyl Glycidyl Ether

Verena Müller,[a] Rebecca Matthes,[a] Manfred Wagner,[b] Matthias Bros,[c] Philip Dreier*,[a] and Holger Frey*,[a]

[a.] *Department of Chemistry, Johannes Gutenberg University, Duesbergweg 10-14, D-55128 Mainz*
[b.] *Max Planck Institute for Polymer Chemistry, Ackermannweg 10, D-55128 Mainz*
[c.] *University Medical Centre, Johannes Gutenberg University, Langenbeckstraße 1, D-55101 Mainz.*

Content

1. Jaacks method for calculation of the reactivity ratios
2. Synthesis of the monomer ethoxy ethyl glycidyl ether (EEGE)
3. NMR characterization of P(EEGE-*co*-EGE)
4. Calculation of copolymer composition and molecular weight
5. SEC data for of P(EEGE-*co*-EGE)
6. MALDI-ToF MS characterization of P(EEGE-*co*-EGE)
7. NMR characterization of P(*lin*G-*co*-EGE)
8. SEC data for P(*lin*G-*co*-EGE)
9. MALDI-ToF MS characterization of P(*lin*G-*co*-EGE)
10. Determination of the reactivity ratios r_{EEGE} and r_{EGE}
11. Investigation of the critical solution behavior of P(*lin*G-*co*-EGE) via turbidimetry
12. Investigation of immune cell viability and immunophenotype of P(*lin*G-*co*-EGE)

1. Jaacks method for calculation of the reactivity ratios

The Jaacks method is a simplification of the well-known Mayo-Lewis equation[1] (Eq. S1) for the copolymerization of the monomers M_1 and M_2.

$$\frac{d[M_1]}{d[M_2]} = \frac{r_1 \frac{[M_1]}{[M_2]} + 1}{r_2 \frac{[M_2]}{[M_1]} + 1} \quad (S1)$$

with $r_1 = \frac{k_{11}}{k_{12}}$ and $r_2 = \frac{k_{22}}{k_{21}}$. The simplification of the Jaacks method is based on the use of an excess of M_1. Therefore, the active chain ends consist almost completely of M_1 units, and the active chain ends with M_2 units can approximately be neglected.[2] The rates of monomer consumption can be simplified as followed:

$$\frac{-\,d[M_1]}{dt} = k_{11}[M_1][P_1^*] \quad (S2)$$

$$\frac{-\,d[M_2]}{dt} = k_{12}[M_2][P_1^*] \quad (S3)$$

where P_1^* is the active chain end with a M_1 unit. The division of Eq. S2 and Eq. S3 results in

$$\frac{d[M_1]}{d[M_2]} = r_1 \frac{[M_1]}{[M_2]} \quad (S4)$$

which can also be obtained from Eq. S1 for $r_1 \frac{[M_1]}{[M_2]} \gg 1$ and $r_2 \frac{[M_2]}{[M_1]} \ll 1$.[2] Integration of Eq. S4 yields Eq. S5.

$$\log\left(\frac{[M_1]_t}{[M_1]_0}\right) = r_1 \cdot \log\left(\frac{[M_2]_t}{[M_2]_0}\right). \quad (S5)$$

The integrated equation (Eq. S5) is valid also for high conversions, if the excess of M_1 over M_2 is ensured.[2] In a plot of $\log\left(\frac{[M_1]_t}{[M_1]_0}\right)$ vs. $\log\left(\frac{[M_2]_t}{[M_2]_0}\right)$, r_1 can be obtained as the slope of the graph. For non-terminal models, r_2 can be calculated from r_1, since $r_1 \cdot r_2 = 1$.[2,3]

2. Synthesis of the monomer ethoxy ethyl glycidyl ether (EEGE)

The monomer EEGE was synthesized according to a literature synthesis by Fitton *et al.*[4] Fig. S1 shows the ^{1}H NMR spectrum of the synthesized EEGE.

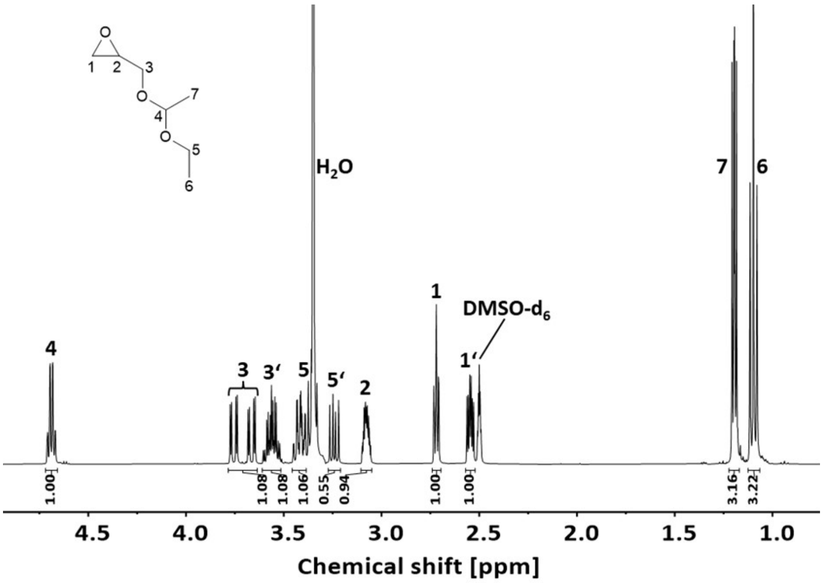

Fig. S1: H NMR spectrum (400 MHz, DMSO-d$_6$) of the synthesized monomer EEGE.

3. NMR characterization of P(EEGE-*co*-EGE)

The ^{1}H NMR spectra of all synthesized homo- and copolymers are shown in Fig. S2, the ^{13}C NMR spectra are summarized in Fig. S3.

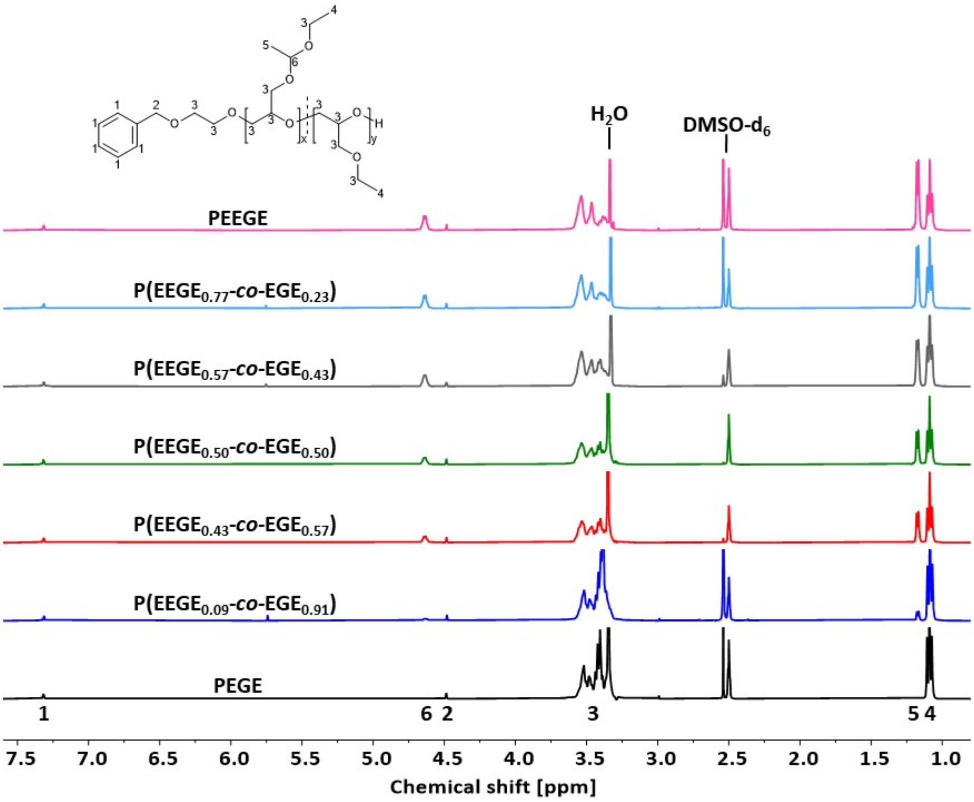

Fig. S2: Stacked ^{1}H NMR spectra (400 MHz, DMSO-d$_6$) of combined P(EEGE-*co*-EGE) copolymers and the homopolymers PEGE and PEEGE.

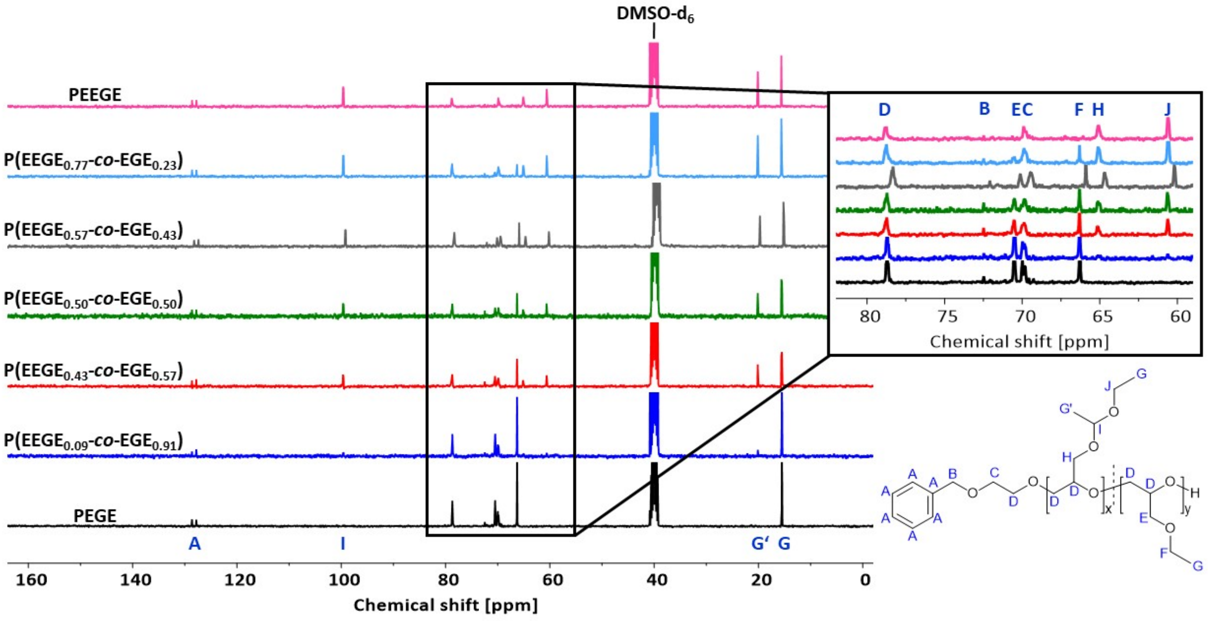

Fig. S3: Stacked ^{13}C NMR spectra (100 MHz, DMSO-d_6) of combined P(EEGE-*co*-EGE) copolymers and the homopolymers PEGE and PEEGE.

4. Calculation of copolymer composition and molecular weight

Calculation of the copolymer composition is exemplary shown for sample P($EEGE_{0.43}$-*co*-$EGE_{0.57}$) (Fig. S4).

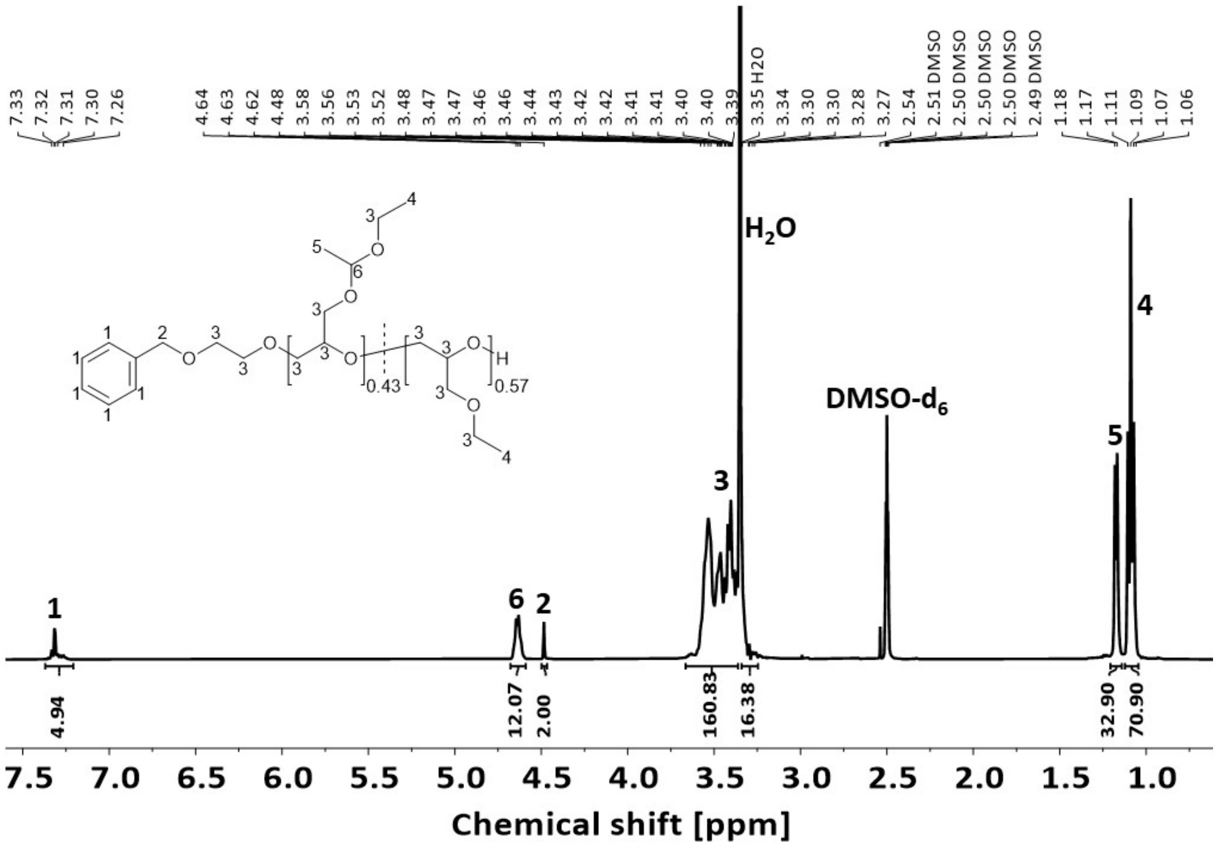

Fig. S4: ^{1}H NMR spectrum (400 MHz, DMSO-d_6) of P($EEGE_{0.43}$-*co*-$EGE_{0.57}$).

The spectrum was referenced to the solvent signal (DMSO-d_6, δ = 2.50 ppm). The integral of the methylene group of the initiator end group (integral 2) was defined as 2. The degree of polymerization of EEGE (DP_{EEGE}) is calculated by division of integral 5 by 3 protons of the methyl group (H5). For the calculation of the degree of polymerization of EGE (DP_{EGE}), the number of protons of integral 5 is subtracted from integral 4. DP_{EGE} is then calculated by division by 3 protons of the methyl group (H4) (Eq. S6).

$$DP_{EGE} = \frac{\text{integral 4 - integral 5}}{3} \tag{S6}$$

The molecular weight is calculated by multiplying the degree of polymerization of the monomers with the corresponding molar mass and addition of the molar mass of the end groups.

5. SEC data for P(EEGE-*co*-EGE)

SEC measurements were performed in DMF and toluene as internal standard and calibrated with PEG standards. The SEC curves of additional P(EEGE-*co*-EGE) copolymers are shown in Fig. S5. The molecular weight distributions are narrow and monomodal for all copolymers.

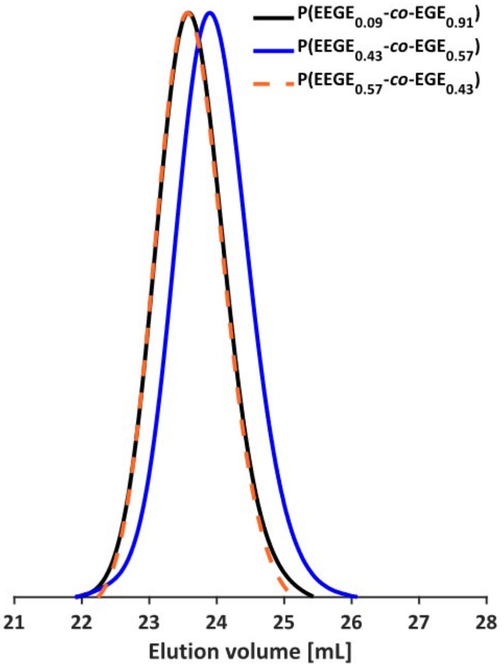

Fig. S5: Additional SEC curves (DMF, PEG calibration) of P(EEGE-*co*-EGE) copolymers.

6. MALDI-ToF MS characterization of P(EEGE-*co*-EGE)

An enlargement of the MALDI-ToF mass spectrum of P($EEGE_{0.57}$-*co*-$EGE_{0.43}$) (see Fig. 1) is shown in Fig. S6 where a glycidol repeating unit is highlighted. Parts of the acetal protecting groups of the EEGE units were deprotected by the acidic salt additive trifluoroacetic acid potassium salt during the measurement.

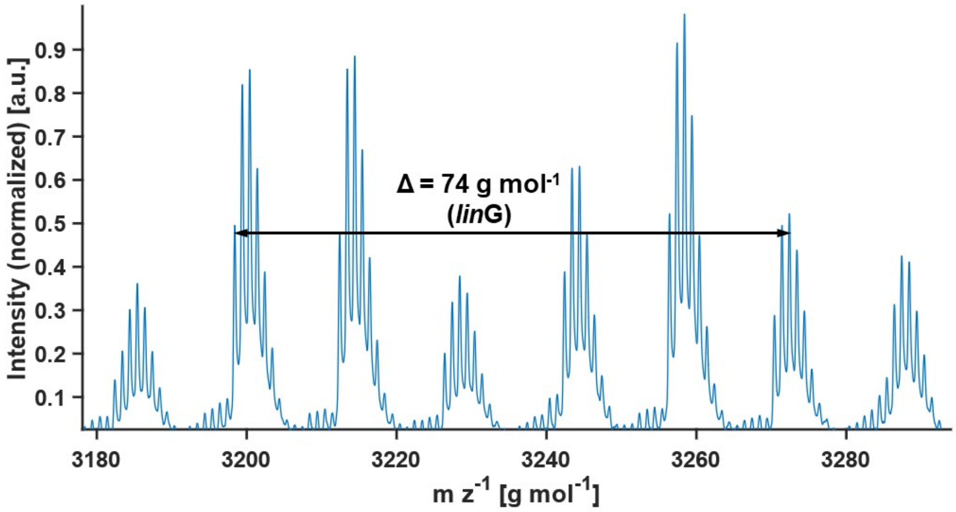

Fig. S6: Enlargement of the MALDI-ToF mass spectrum of P(*lin*$G_{0.57}$-*co*-$EGE_{0.43}$). The repeating unit of the deprotected glycidol is highlighted. Matrix: *trans*-2-[3-(4-*tert*-butylphenyl)-2-methyl-2-propenyliden]-malononitrile (DCTB), salt additive: trifluoroacetic acid potassium salt.

7. NMR characterization of P(*lin*G-*co*-EGE)

The ^{1}H NMR spectra of all deprotected homo- and copolymers are shown in Fig. S7, the ^{13}C NMR spectra are summarized in Fig. S8. The spectra display no acetal proton signals, the cleavage of the EEGE acetal protecting groups has been successful.

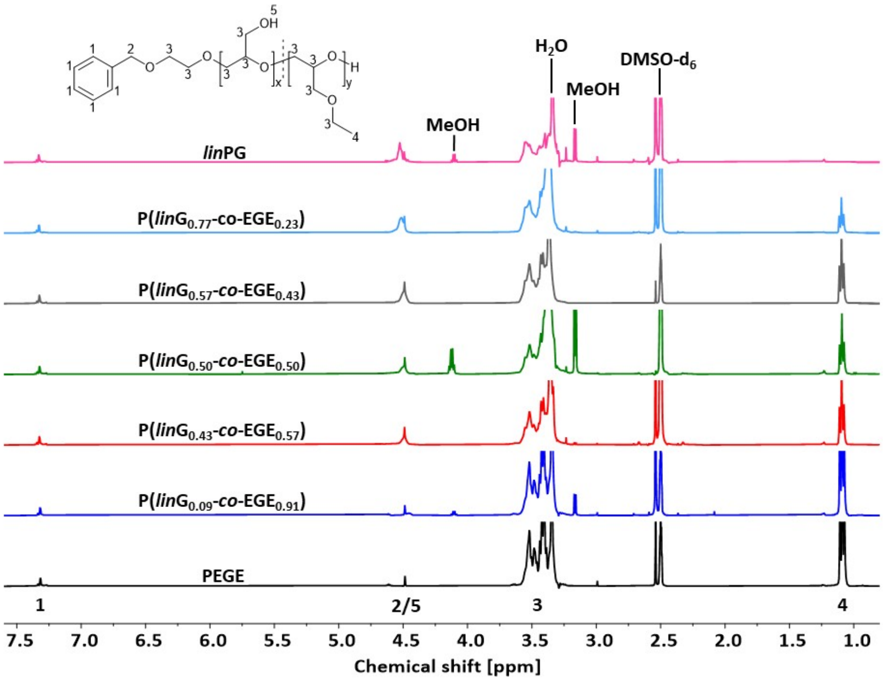

Fig. S7: Stacked ^{1}H NMR spectra (400 MHz, DMSO-d_6) of combined P(*lin*G-*co*-EGE) copolymers and the homopolymers PEGE and *lin*PG.

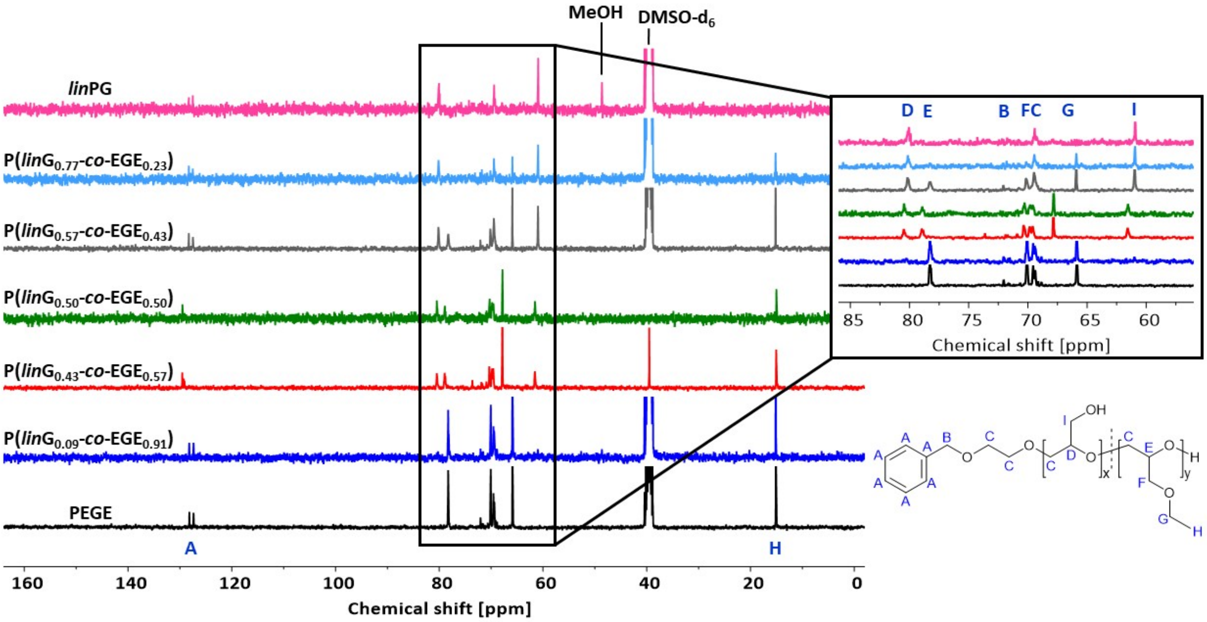

Fig. S8: Stacked ^{13}C NMR spectra (100 MHz, DMSO-d$_6$) of combined P(*lin*G-*co*-EGE) copolymers and the homopolymers PEGE and *lin*PG.

8. SEC data for P(*lin*G-*co*-EGE)

The SEC curves of additional P(*lin*G-*co*-EGE) copolymers are shown in Fig. S9. The molecular weight distributions are narrow and monomodal for all copolymers but P(*lin*G$_{0.57}$-*co*-EGE$_{0.43}$). The elugram of this copolymer is broadened towards lower elution volume which is possibly caused by aggregation during the SEC measurement since no allylic initiation is observed in the ^{1}H NMR spectrum and the MALDI-ToF mass spectrum (see Fig. S11, ESI).

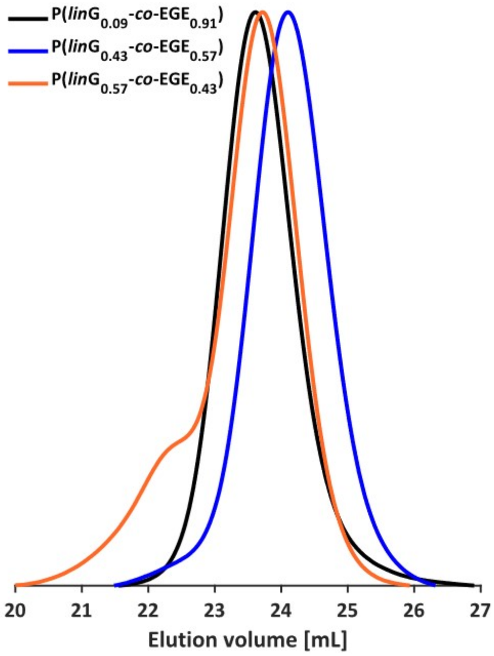

Fig. S9: Additional SEC curves (DMF, PEG calibration) of P(*lin*G-*co*-EGE) copolymers.

Fig. S10 shows the shift in elution volume of the deprotected P(*lin*$G_{0.77}$-*co*-$EGE_{0.23}$) copolymer compared to the protected P($EEGE_{0.77}$-*co*-$EGE_{0.23}$) copolymer. Due to the abstraction of the acetal protecting group, the molecular weight of the deprotected copolymer P(*lin*$G_{0.77}$-*co*-$EGE_{0.23}$) decreases during the deprotection and therefore the elution volume increases.

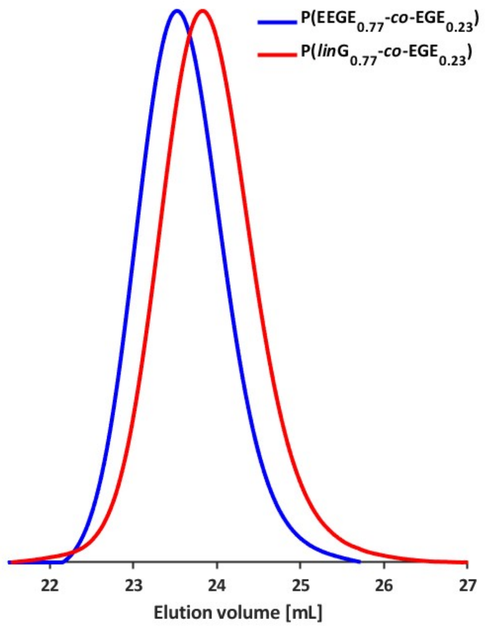

Fig. S10: Comparison of SEC traces (DMF, PEG calibration) of the protected P($EEGE_{0.77}$-*co*-$EGE_{0.23}$) copolymer and the unprotected P(*lin*$G_{0.77}$-*co*-$EGE_{0.23}$) copolymer.

9. MALDI-ToF MS characterization of P(*lin*G-*co*-EGE)

All P(*lin*G-*co*-EGE) copolymers and the homopolymers PEGE and *lin*PG were characterized via MALDI-ToF MS. For all samples but *lin*PG, DCTB was chosen as matrix and KTFA as salt additive. The matrix of *lin*PG was α-cyano-4-hydroxycinnamic acid (HCCA) and lithium chloride was added as salt additive. Fig. S11 shows an exemplary MALDI-ToF mass spectrum of the copolymer P(*lin*$G_{0.57}$-*co*-$EGE_{0.43}$).

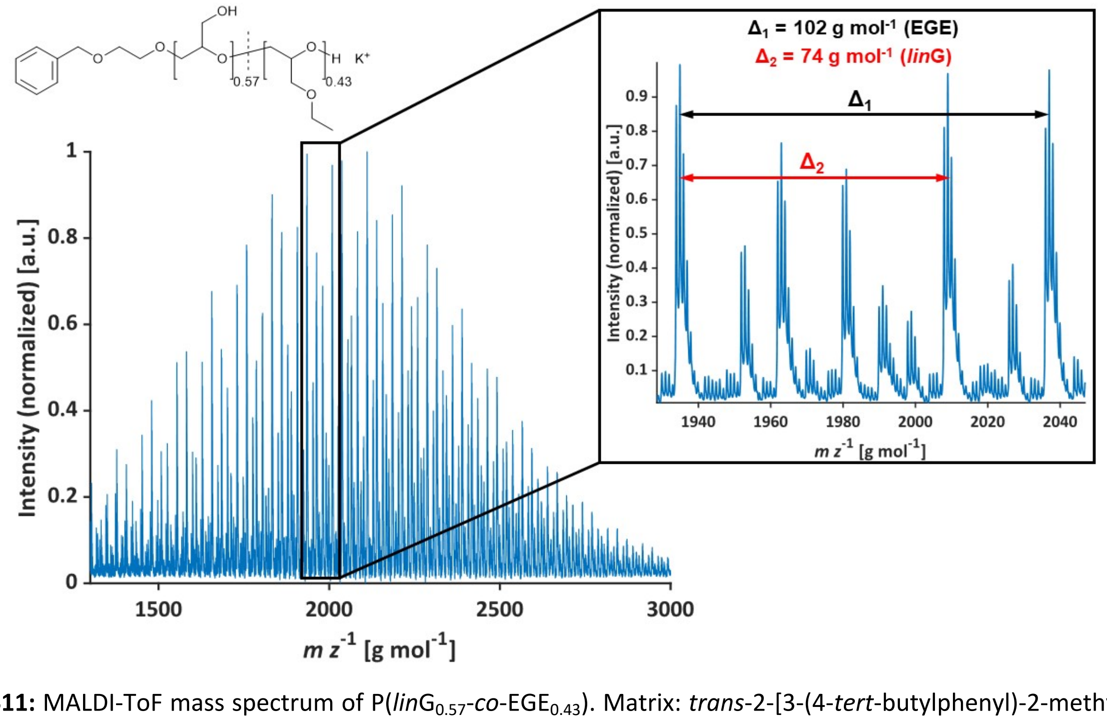

Fig. S11: MALDI-ToF mass spectrum of P(*lin*$G_{0.57}$-*co*-$EGE_{0.43}$). Matrix: *trans*-2-[3-(4-*tert*-butylphenyl)-2-methyl-2-propenyliden]malononitrile (DCTB), salt additive: trifluoroacetic acid potassium salt.

10. Determination of the reactivity ratios r_{EEGE} and r_{EGE}

The monomer consumption was detected by the decrease of the proton signals in the ^{1}H NMR spectra. Since the chemical shifts of the monomer proton signals of EGE and EEGE are similar, only the non-overlapping part of one proton signal of the epoxide methylene group of each monomer is considered (red frame in Fig. S12).

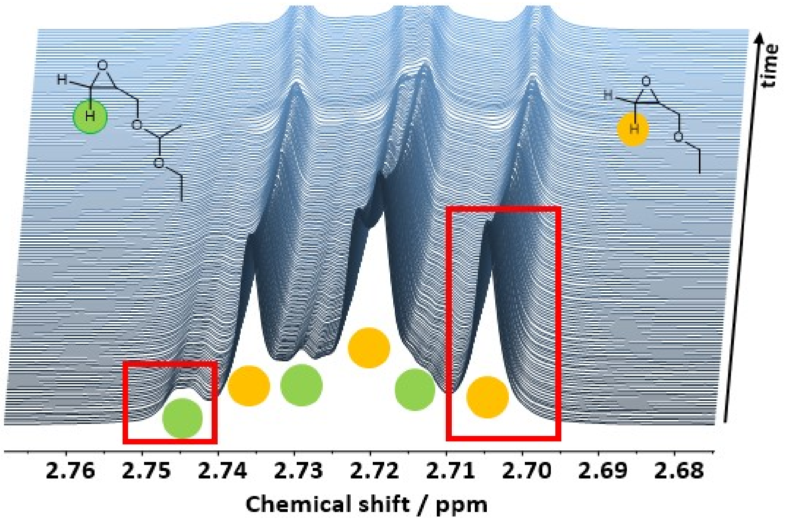

Fig. S12: *In situ* ^{1}H NMR kinetics study (300 MHz, DMSO-d_6) of the anionic copolymerization of EEGE and EGE at 25°C.

11. Investigation of the critical solution behavior of P(*lin*G-*co*-EGE) via turbidimetry

The data points of the turbidimetry vs. temperature curves were fitted by the following equation:

$$y = A_1 + (A_2 - A_1) \cdot \left[\frac{p}{1 + 10^{(\log(m_1 - x)h_1)}} + \frac{1 - p}{1 + 10^{(\log(m_2 - x)h_2)}}\right]. \quad (S7)$$

The cloud point temperature T_{cp} is defined as the temperature with 50% transmittance.

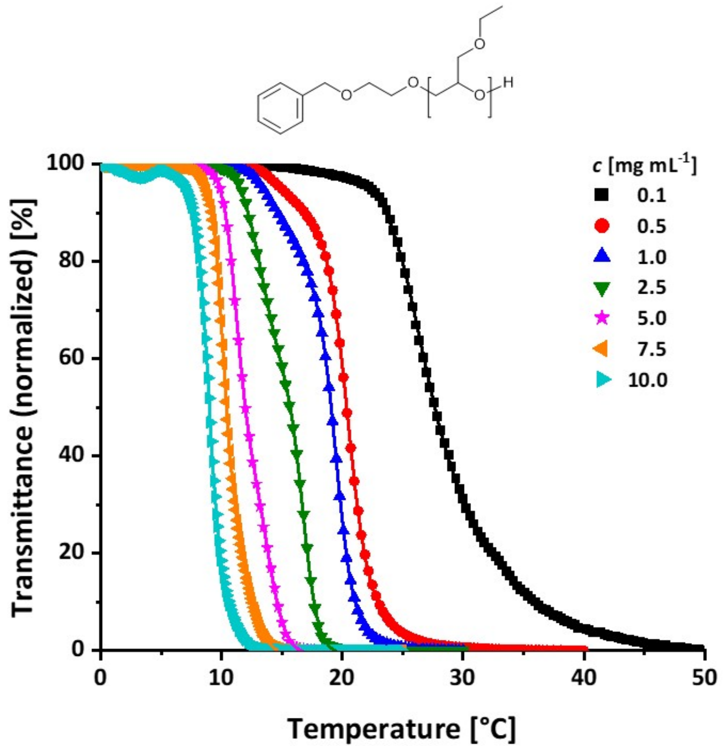

Fig. S13: Transmittance vs. temperature plot of the homopolymer PEGE. Straight lines shows sigmoidal fit to determine T_{cp} at 50% transmittance.

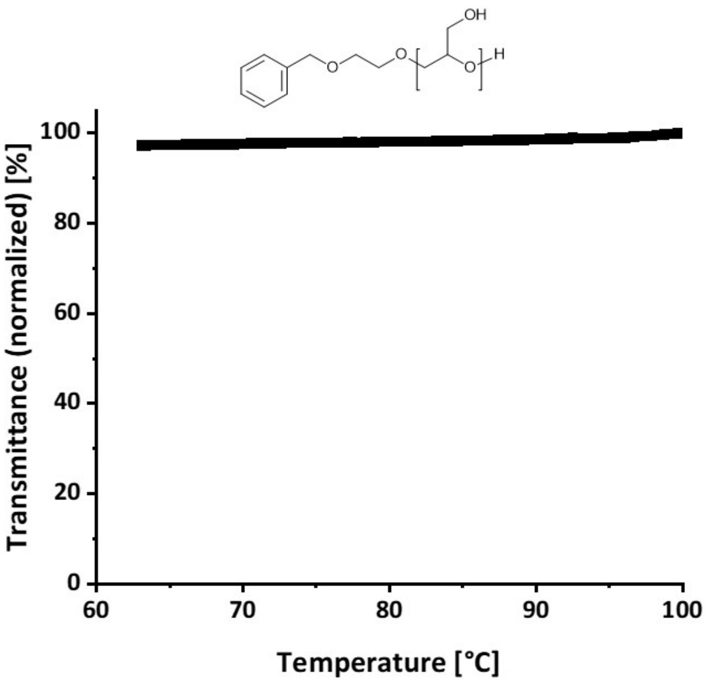

Fig. S14: Transmittance vs. temperature plot of the homopolymer *lin*PG (c = 10.0 mg mL^{-1}). There is no change in transmittance, the T_{cp} of *lin*PG is above the measurable temperature area of water.

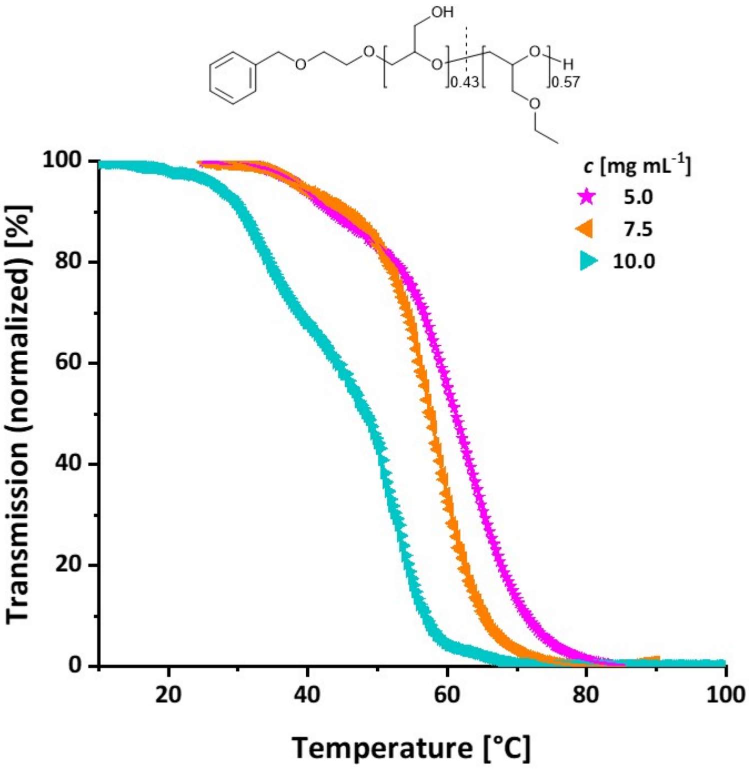

Fig. S15: Transmittance vs. temperature plot of the copolymer P(*lin*$G_{0.43}$-*co*-$EGE_{0.57}$). Straight lines shows sigmoidal fit to determine T_{cp} at 50% transmittance.

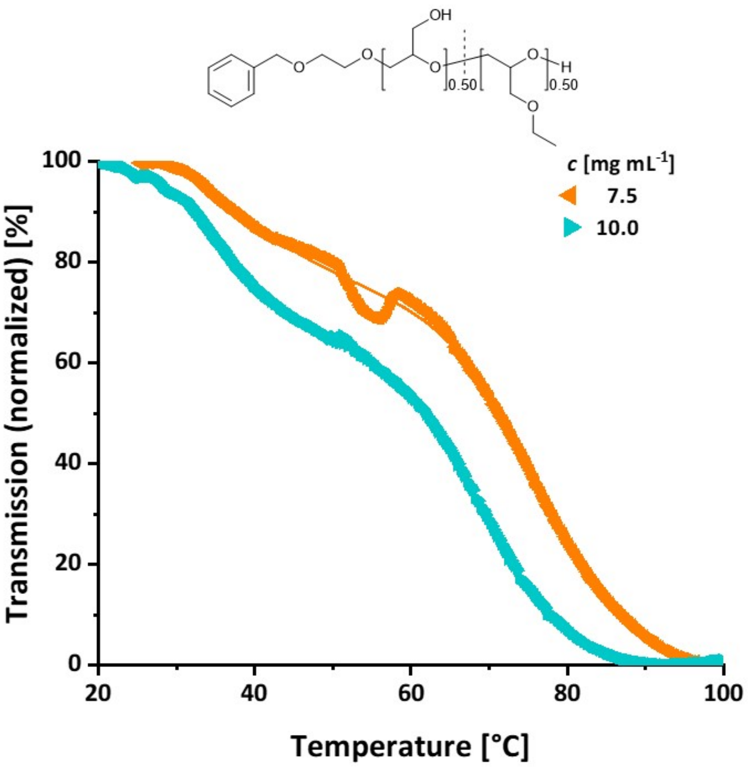

Fig. S16: Transmittance vs. temperature plot of the copolymer P(*lin*$G_{0.50}$-*co*-$EGE_{0.50}$). Straight lines shows sigmoidal fit to determine T_{cp} at 50% transmittance.

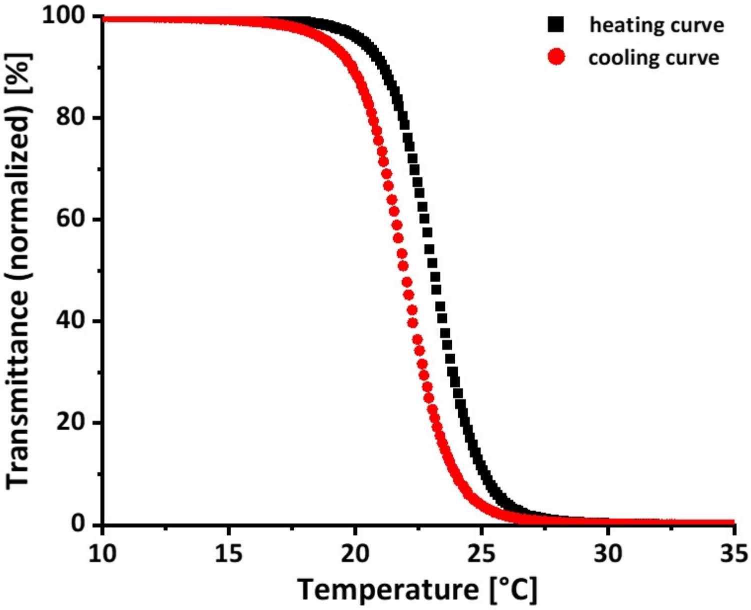

Fig. S17: Transmittance vs. temperature plot of heating and cooling curves of the copolymer P(*lin*$G_{0.09}$-*co*-$EGE_{0.91}$) with a concentration of c = 2.5 mg mL^{-1} showing a negligibly small hysteresis.

12. Investigation of immune cell viability and immunophenotype of P(*lin*G-*co*-EGE)

The cell viability of P(*lin*$G_{0.57}$-*co*-$EGE_{0.43}$) and the reference monomethyl poly(ethylene glycol) (mPEG) was investigated via fluorescence-activated cell scanning (FACS) for dendritic cells (DC), polymorphonuclear cells (PMN) and T cells, the immunology was investigated via expression of the surface protein CD80 for dendritic cells (DC) and polymorphonuclear cells (PMN), as shown in Fig. S18.

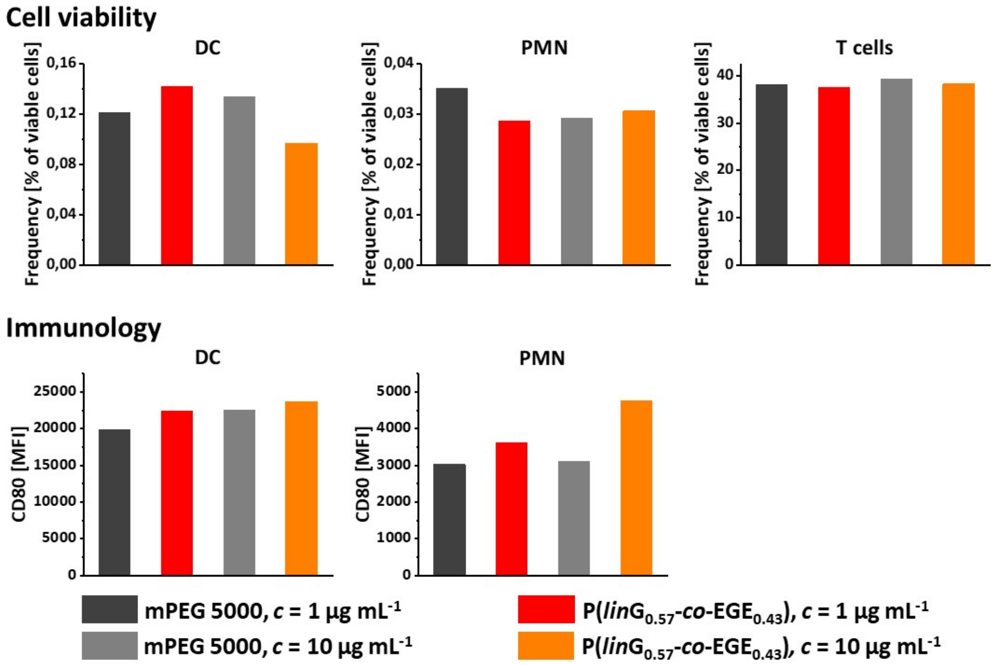

Fig. S18: Top: Cell viability of P(*lin*$G_{0.57}$-*CO*-$EGE_{0.43}$) and mPEG (M_n = 5000 g mol^{-1}) as a reference for dendritic cells (DC), polymorphonuclear cells (PMN) and T cells. Bottom: Immunology of P(*lin*$G_{0.57}$-*CO*-$EGE_{0.43}$) and mPEG (M_n = 5000 g mol^{-1}) as a reference for dendritic cells (DC) and polymorphonuclear cells (PMN) using the surface protein CD80 (MFI: mean fluorescence intensity).

Further immunology tests were carried out via expression of the surface proteins CD86 (Fig. S19, top) and MHCII (Fig. S19, bottom) for the following cell lines: B cells, dendritic cells (DC), natural killer cells (NK), macrophages and polymorphonuclear cells (PMN).

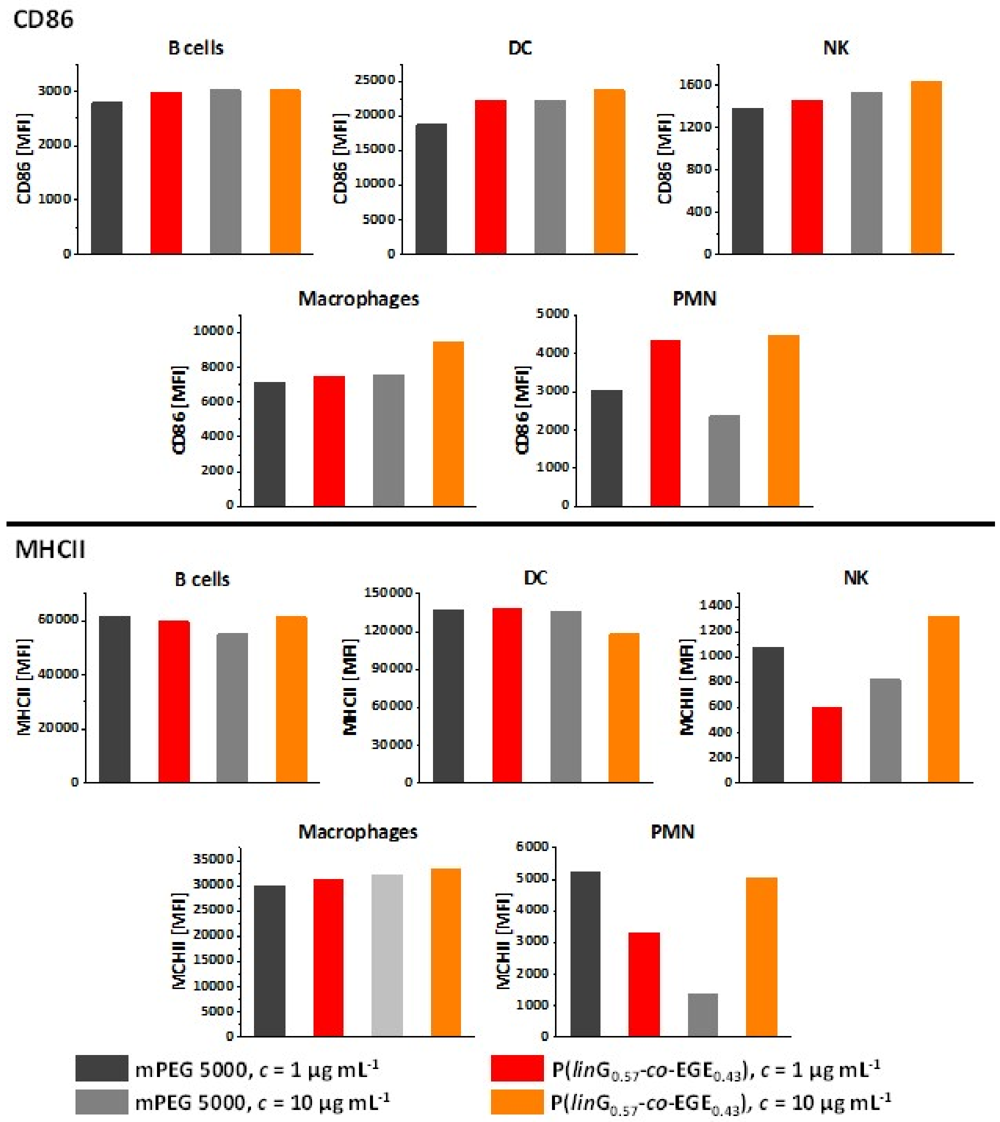

Fig. S19: Immunology of P(*lin*$G_{0.57}$-*co*-$EGE_{0.43}$) and mPEG (M_n = 5000 g mol^{-1}) as a reference for several cell lines using the surface proteins CD86 (top) and MCHII (bottom).

References

1 F. R. Mayo and F. M. Lewis, *J. Am. Chem. Soc*, 1944, **66**, 1594.

2 V. Jaacks, *Makromol. Chem.*, 1972, **161**, 161–172.

3 F. T. Wall, *J. Am. Chem. Soc*, 1944, **66**, 2050.

4 A. O. Fitton, J. Hill, D. E. Jane, R. Millar, *Synthesis*, 1987, **12**, 1140–1142.
